# Supplementary material for: Associations between multimorbidity and adverse health outcomes in UK Biobank and the SAIL Databank: A comparison of longitudinal cohort studies
Source: PLoS Med. 2022 Mar 7;19(3):e1003931. doi: 10.1371/journal.pmed.1003931 (PMC8901063; doi:10.1371/journal.pmed.1003931)
Supplement: S1 Protocol — (DOCX) [file pmed.1003931.s001.docx]

UK Biobank and SAIL comparison: Multimorbidity prevalence and relationship with mortality and hospitalization

# Background

UK Biobank research cohort is a large sample of volunteers recruited between 2006 and 2010 across England, Scotland and Wales.

It is well established that UK Biobank is not representative of the UK population.^1^ Participants are, on average, more affluent, more likely to be of white ethnicity, and have a lower prevalence of a number of chronic health conditions.^1^ While UK Biobank have claimed that inferences about the assocaitions between exposure and outcomes “not require participants to be representative of the population”, others have questioned this assertion and highlighted that selection bias may introduce spurious relationships between exposures and outcomes.^2^

UK Biobank has proved to be a useful and popular resource for assessing the impact of multimorbidity.^3-7^ However, these studies have not explored the impact of the representativeness (or lack thereof) of UK Biobank on the relationships between multimorbidity and clinical outcomes.

# Research question

1. How does the distribution of multimorbidity in UK Biobank differ from a nationally representative sample (using Secure Anonymized Information Datalink (SAIL) databank)
2. Do differences in the socioeconomic deprivation between UK Biobank and SAIL account for these differences
3. How does the relationship between multimorbidity and adverse health outcomes (mortality and hospitalization) differ between UK Biobank and SAIL

# Aims

## Research question 1 and 2 - representativeness

1. Describe the distribution of multimorbidity (based on a count of LTCs) in UK Biobank and in SAIL
2. Calculate the expected distribution of multimorbidity in UK Biobank based on SAIL data:
   1. After standardizing SAIL to the age/sex distribution of UK Biobank
   2. After standardizing SAIL to the age/sex/socioeconomic status distribution of UK Biobank
3. Compare the observed distribution on multimorbidity in UK Biobank to the expected distribution.

This will assess to what extent UK Biobank is under-representative of multimorbidity, and to what extent this is driven by differences in socioeconomic status.

## Research question 3 – association with outcomes

1. Fit a model in SAIL to describe the relationship between multimorbidity and:
   1. All-cause mortality
   2. Hospitalisation
2. Fit a similar model in UK Biobank to assess these same relationships
3. Compare the relationship between multimorbidity and mortality/hospitalization between the two datasets

# Data sources

UK Biobank data will be used from UK Biobank project 14151. We will analyse the subset of participants for whom primary care data have been linked and made available to researchers (n=230,105 people) to allow a consistent definition of multimorbidity to be applied across the two datasets.

For a nationally representative population we well use data from the SAIL databank under project 0830. This data is available for analysis through a secure portal and contains linked primary care records, hospitalization data, and mortality records. We will limit our sample to people registered with a participating practice throughout 2011 (to coincide with the completion of UK Biobank recruitment and with relative completeness of read-code data within the dataset). We will also limit the sample to people aged 40-70 years (intended recruitment to UK Biobank)

# Covariates

Age:

- Age at baseline UK Biobank assessment centre for UK Biobank
- Age on 1^st^ January 2011 for SAIL

Sex

- Coded 1 = male, 2 = female in both datasets

Socioeconomic status

- UK Biobank baseline data are linked to Townsend scored based on participant postcodes linked to previous (2001) census data. This gives an area-based measure of socioeconomic status
- For SAIL, data is available for Lower Layer Super Output Area (LSOA) for each individual. Data on Towsend scores based on the same (2001) census data can therefore be linked using publicly available data on LSOA and electoral Wards from the Office of National Statistics.
- Townsend will be used in favour of the Welsh Index of Multiple Deprivation as this is not directly comparable, and on a different scale, from the Index of Multiple Deprivation used in England, for which UK Biobank data are available.

Multimorbidity

- Multimorbidity will be assessed using a count of long-term conditions (LTCs) (n=40) from previous population-based studies of multimorbidity. Each condition will be identified using Read codes occurring prior to the UK Biobank assessment centre or prior to 2011 for UK Biobank and SAIL, respectively. Read-code definition will be identical between datasets, and based on definitions described by Barnett et al.^8^
- For subsequent analysis using the weighted ‘Cambridge Multimorbidity Score’ (described under ‘exploring assumptions and explanations’) we will use the same read-coded definitions of the relevant LTCs and assign weights as per the validation of the Cambridge Multimorbidity Score.^9^

# Analysis

For illustrative purposes, the analysis plan below presents real data/outputs from UK Biobank alongside hypothetical findings from SAIL. This is to illustrate how the findings might be presented, however these are not the results of the comparison analysis (which has not yet been performed)

## Distribution of multimorbidity

The UK Biobank dataset is downloaded and available to analyse directly.

SAIL, in contrast, is held within a secure repository and allows only export of aggregate, non-disclosive information, model outputs etc.

To compare the distribution of multimorbidity between UK Biobank and SAIL, we will:

1. Plot the observed distribution of LTC counts using the UK Biobank dataset (grey bars on example plot 1)
2. Plot a similar distribution for SAIL within the secure environment.
3. Fit a count-based model (e.g. poisson, negative binomial) to the distribution in SAIL to describe the distribution. Test this graphically and with statistical test (e.g. Kolmogorov-Smirnoff test) within the secure environment.
4. Fit a further 2 models to the SAIL data modelling the distribution of LTC count on and age sex (model 1) and on age, sex, and Townsend score (model 2).
5. We will then test the fit of the model at the range of observed values of age, sex, and Townsend scores, and compare the modelled distribution to the observed distribution of counts stratified by age, sex and quintiles of the Townsend score. This should result in a model which can accurately describe the distribution of multimorbidity conditional on age, sex and socioeconomic status within the SAIL dataset.
6. Export this model, along with aggregate data describing the raw counts, from SAIL
7. Using the coefficients from model 1, we will calculate expected values for LTC counts for each combination of age and sex in the UK Biobank dataset. We will then multiply these by the relative frequency of each age/sex combination, and sum across these to obtain an expected count of LTCs for UK Biobank standardized by age and sex. This will be plotted against the observed distribution to assess the representativeness of UK Biobank conditional on age and sex (hypothetical example shown in the blue line in example plot 1).
8. We will repeat this process with model 2, obtaining expected counts for each level of age/sex/socioeconomic status. Summing across these values as before will obtain an expected distribution for UK Biobank standardized by age, sex and deprivation (hypothetical data shown in red line in example plot 1).

| Example plot 1 |
| --- |
| 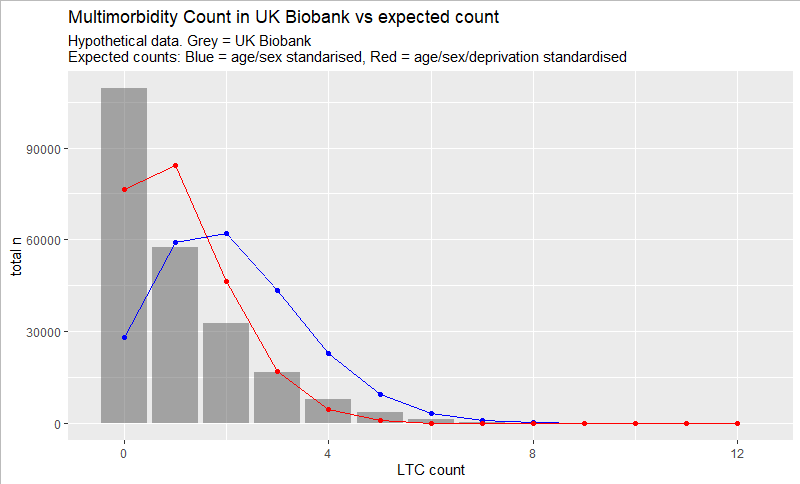 |

## Association between multimorbidity and outcomes

Having explored the representativeness of UK Biobank in terms of LTC counts, we will then assess if the relationship between LTC counts and clinical outcomes (mortality and hospitalization) are similar between UK Biobank and SAIL.

Given the known under-representativeness of UK Biobank in general, we hypothesize that multimorbidity will be under-represented. These subsequent analyses will explore whether, despite this under-representativeness, multimorbidity identified in UK Biobank is associated with a similar level of risk of adverse health outcomes to multimorbidity identified in SAIL.

We will use the flexsurv package in R to fit parametric survival models (e.g. Weibull, Generalised gamma etc.) for the analysis of all-cause mortality.

For hospitalization events, we will fit a count-based model (e.g. poisson, negative binomial etc.).

The analysis process will be as follows (taking mortality as the example):

1. Fit a model to the SAIL data modelling time to death on age, sex, Townsend score and LTC count.
2. Explore non-linear associations using fractional polynomials, and assess interaction terms between these covariates.
3. Select a best-fitting model using e.g. model AIC and assess the fit of this model to the SAIL data graphically (e.g. <https://cran.r-project.org/web/packages/flexsurv/vignettes/flexsurv.pdf>
4. Export the model coefficients and variance covariance matrix from the secure environment.
5. Outside the secure environment, plot the modelled 5-year mortality for given age/sex/deprivation/multimorbidity combinations (e.g. red lines on example plots 2 and 3 – hypothetical data only)
6. Fit a similar model to the UK Biobank dataset, and obtain estimates of 5-year mortality for the same covariate levels. Plot these alongside the estimates from SAIL (e.g. blue lines on example plots 2 and 3)

| Example plot 2 –possible results under scenario a) that the association between multimorbidity and mortality is similar between UK Biobank and SAIL and a given age/sex/socioeconomic status |
| --- |
| 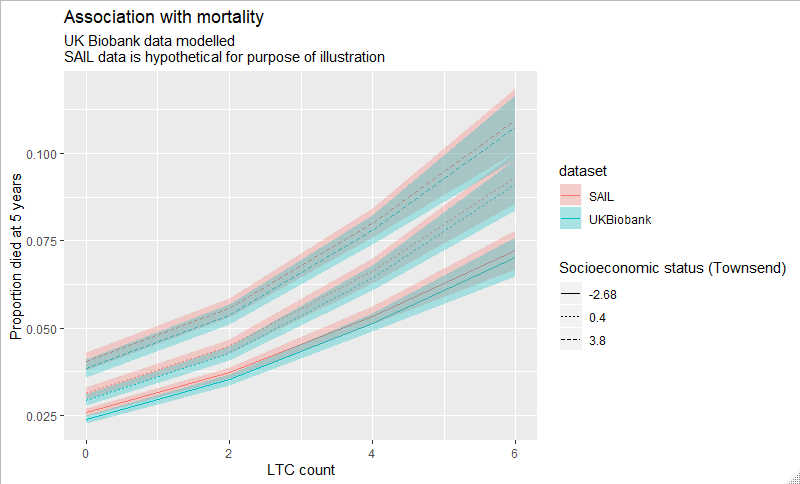 |
| Example plot 3 – possible results under scenario b) that at a given level of multimorbidity/deprivation, UK Biobank participants may have a different risk of mortality than people identified in SAIL |
| 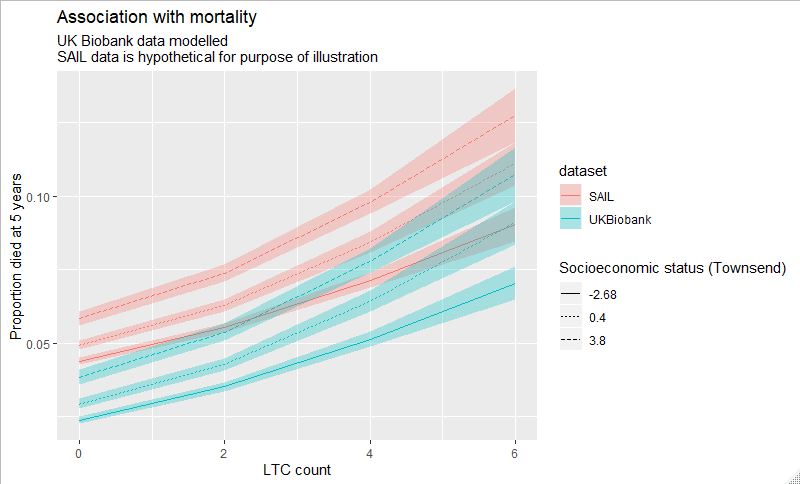 |

This analysis assessing outcomes will explore two possible scenarios:

1. UK Biobank is under-representative in terms of multimorbidity and socioeconomic deprivation, however the association with mortality at a given level of multimorbidity and deprivation is similar between UK Biobank and SAIL (in which case result may resemble example plot 2, above)
2. Or, at a given level of multimorbidity/deprivation, UK Biobank participants may have a different risk of mortality than people identified in SAIL (e.g. example plot 3)

We will then calculate the hazard ratios for various levels of LTC count (1, 2, 3, 4, 5 and 6 LTCs) using 0 LTCs as a reference category. These will be derived from the UK Biobank and the SAIL models separately. Where interaction terms have been included, we will assess hazard ratios at the mean age and Townsend score for the UK Biobank dataset. We will then compare the hazard ratios between UK Biobank and SAIL to assess whether the *relative* difference in mortality between LTC counts if different between UK Biobank and SAIL.

# Exploring assumptions and explanations

Unpicking the causal relationships between multimorbidity and outcomes is challenging, particularly as multimorbidity is a composite measure of a wide range of LTCs.

Each of these LTCs will have a different range of predisposing factors (although some of these may be shared), which may in turn have their own independent relationships with mortality.

If the results of the analyses resemble scenario B – that the association with mortality/hospitalization at a given level of multimorbidity is different between UK Biobank and SAIL – this could have several possible explanations:

1. The *type* of long-term conditions differs between UK Biobank and SAIL
2. The *severity* of the underlying conditions that make up the LTC count differs between UK Biobank and SAIL
3. Other factors (e.g. behavioural risk factors) differ between UK Biobank and SAIL and impact mortality independently of multimorbidity

With the available data in UK Biobank and SAIL, explanation 1 can be readily explored, whereas 2 and 3 cannot be reliably assessed in both datasets using similar approaches.

To assess differences in type of LTC between UK Biobank and SAIL, we will use 3 approaches:

- Plot the prevalence of each of the 40 LTCs that make up the count and compare the prevalence between UK Biobank and SAIL (descriptive assessment of LTC type only)
- Use a weighted score to assess multimorbidity. For this we will use the Cambridge Multimorbidity score as it is validating using primary care data to identify multimorbidity (using read codes as we will in this study). It also includes validated weights specific to mortality and to hospitalization. We will therefore repeat the analysis of outcomes, as described above, using a weighted score instead of a simple count. If it is true that differences between UK Biobank and SAIL are explained by differences in the type of LTCs, we would expect using a weighted score to attenuate these differences.
- Identify specific clusters of LTCs in both datasets and assess their relationship with mortality. A recent study using CPRD data identified age-specific clusters of LTCs in people with multimorbidity what were associated with mortality and healthcare utilization. We will therefore compare the prevalence of each cluster between the two datasets, and the rate of mortality and hospitalization within that cluster of LTCs. If any differences between UK Biobank and SAIL are driven by differences in type of LTCs, then we would expect the rates within specific clusters to be similar. If, however, event rates differ between dataset for similar clusters of conditions, this would suggest that severity or other risk factors are likely to underly any residual observed differences.^10^

# References

1. Fry A, Littlejohns TJ, Sudlow C, et al. Comparison of sociodemographic and health-related characteristics of UK Biobank participants with those of the general population. *American journal of epidemiology* 2017; **186**(9): 1026-34.

2. Keyes KM, Westreich D. UK Biobank, big data, and the consequences of non-representativeness. *The Lancet* 2019; **393**(10178): 1297.

3. Chudasama YV, Khunti KK, Zaccardi F, et al. Physical activity, multimorbidity, and life expectancy: a UK Biobank longitudinal study. *BMC Med* 2019; **17**(1): 108.

4. Zemedikun DT, Gray LJ, Khunti K, Davies MJ, Dhalwani NN. Patterns of multimorbidity in middle-aged and older adults: an analysis of the UK Biobank data. Mayo Clinic Proceedings; 2018: Elsevier; 2018. p. 857-66.

5. Di Angelantonio E, Kaptoge S, Wormser D, et al. Association of cardiometabolic multimorbidity with mortality. *Jama* 2015; **314**(1): 52-60.

6. Hanlon P, Nicholl BI, Jani BD, Lee D, McQueenie R, Mair FS. Frailty and pre-frailty in middle-aged and older adults and its association with multimorbidity and mortality: a prospective analysis of 493 737 UK Biobank participants. *The Lancet Public health* 2018; **3**(7): e323-e32.

7. Jani BD, Hanlon P, Nicholl BI, et al. Relationship between multimorbidity, demographic factors and mortality: findings from the UK Biobank cohort. *BMC Med* 2019; **17**(1): 74.

8. Barnett K, Mercer SW, Norbury M, Watt G, Wyke S, Guthrie B. Epidemiology of multimorbidity and implications for health care, research, and medical education: a cross-sectional study. *The Lancet* 2012; **380**(9836): 37-43.

9. Payne RA, Mendonca SC, Elliott MN, et al. Development and validation of the Cambridge Multimorbidity Score. *CMAJ* 2020; **192**(5): E107-E14.

10. Zhu Y, Edwards D, Mant J, Payne RA, Kiddle S. Characteristics, service use and mortality of clusters of multimorbid patients in England: a population-based study. *BMC Med* 2020; **18**: 1-11.
