# Supplementary material for: Associations between multimorbidity and adverse health outcomes in UK Biobank and the SAIL Databank: A comparison of longitudinal cohort studies
Source: PLoS Med. 2022 Mar 7;19(3):e1003931. doi: 10.1371/journal.pmed.1003931 (PMC8901063; doi:10.1371/journal.pmed.1003931)
Supplement: S1 Checklist — (DOCX) [file pmed.1003931.s002.docx]

STROBE Statement—Checklist of items that should be included in reports of ***cohort studies***

|  | Item No | Recommendation | Page No |
| --- | --- | --- | --- |
| **Title and abstract** | 1 | (*a*) Indicate the study’s design with a commonly used term in the title or the abstract | Title |
|  |  | (*b*) Provide in the abstract an informative and balanced summary of what was done and what was found | Abstract |
| Introduction | | | |
| Background/rationale | 2 | Explain the scientific background and rationale for the investigation being reported | Intro, para 2-4 |
| Objectives | 3 | State specific objectives, including any prespecified hypotheses | Intro para 5 |
| Methods | | | |
| Study design | 4 | Present key elements of study design early in the paper | Methods para 1 |
| Setting | 5 | Describe the setting, locations, and relevant dates, including periods of recruitment, exposure, follow-up, and data collection | Methods: data sources (all paragraphs) and Outcomes (all paragraphs) |
| Participants | 6 | (*a*) Give the eligibility criteria, and the sources and methods of selection of participants. Describe methods of follow-up | Methods, data sources, para 1 and 3 |
|  |  | (*b*) For matched studies, give matching criteria and number of exposed and unexposed |  |
| Variables | 7 | Clearly define all outcomes, exposures, predictors, potential confounders, and effect modifiers. Give diagnostic criteria, if applicable | Methods: assessment of multimorbidity (para 1-5), covariates (para 1) and outcomes (para 1).  S1 File and S2 file for diagnostic codes. |
| Data sources/ measurement | 8* | For each variable of interest, give sources of data and details of methods of assessment (measurement). Describe comparability of assessment methods if there is more than one group | Methods: assessment of multimorbidity (para 1-5), covariates (para 1) and outcomes (para 1) |
| Bias | 9 | Describe any efforts to address potential sources of bias |  |
| Study size | 10 | Explain how the study size was arrived at | Methods, data sources, paras 1-5 |
| Quantitative variables | 11 | Explain how quantitative variables were handled in the analyses. If applicable, describe which groupings were chosen and why | Methods, assessment of multimorbidity (para 3) and covariates (para 1) |
| Statistical methods | 12 | (*a*) Describe all statistical methods, including those used to control for confounding | Methods, Statistical analyses section paras 2-5, 7-8 |
|  |  | (*b*) Describe any methods used to examine subgroups and interactions | Methods, statistical analysis, comparison of multimorbidity distributions (para 1), modelling all-cause mortality and mace (para 1) and modelling unscheduled hospitalisations (para 1) |
|  |  | (*c*) Explain how missing data were addressed | Methods, statistical analysis, comparison of multimorbidity distributions (para 2) |
|  |  | (*d*) If applicable, explain how loss to follow-up was addressed | Methods, outcomes (para 1) |
|  |  | (*e*) Describe any sensitivity analyses | Methods, statistical analysis, Sensitivity analysis (para 1) |
| Results | | |  |
| Participants | 13* | (a) Report numbers of individuals at each stage of study—eg numbers potentially eligible, examined for eligibility, confirmed eligible, included in the study, completing follow-up, and analysed | Supplementary file S1 text |
|  |  | (b) Give reasons for non-participation at each stage | Supplementary file S1 text |
|  |  | (c) Consider use of a flow diagram | Supplementary file S1 text |
| Descriptive data | 14* | (a) Give characteristics of study participants (eg demographic, clinical, social) and information on exposures and potential confounders | Table 1 |
|  |  | (b) Indicate number of participants with missing data for each variable of interest | Table 1 |
|  |  | (c) Summarise follow-up time (eg, average and total amount) | Results para 1 |
| Outcome data | 15* | Report numbers of outcome events or summary measures over time | Figures 3b, 4b, 5b, 6.  Supplementary table |

| Main results | 16 | (*a*) Give unadjusted estimates and, if applicable, confounder-adjusted estimates and their precision (eg, 95% confidence interval). Make clear which confounders were adjusted for and why they were included |  |
| --- | --- | --- | --- |
|  |  | (*b*) Report category boundaries when continuous variables were categorized | Figures 3b, 4b, 5b |
|  |  | (*c*) If relevant, consider translating estimates of relative risk into absolute risk for a meaningful time period | Figures 3a, 4a, 5a |
| Other analyses | 17 | Report other analyses done—eg analyses of subgroups and interactions, and sensitivity analyses | Results, sensitivity analysis, para 1 |
| Discussion | | | |
| Key results | 18 | Summarise key results with reference to study objectives | Discussion para 1 |
| Limitations | 19 | Discuss limitations of the study, taking into account sources of potential bias or imprecision. Discuss both direction and magnitude of any potential bias | Discussion para 11 |
| Interpretation | 20 | Give a cautious overall interpretation of results considering objectives, limitations, multiplicity of analyses, results from similar studies, and other relevant evidence | Discussion para 2-10 |
| Generalisability | 21 | Discuss the generalisability (external validity) of the study results | Discussion para 12 |
| Other information | | | |
| Funding | 22 | Give the source of funding and the role of the funders for the present study and, if applicable, for the original study on which the present article is based | Acknowledgement section |

*Give information separately for exposed and unexposed groups.

**Note:** An Explanation and Elaboration article discusses each checklist item and gives methodological background and published examples of transparent reporting. The STROBE checklist is best used in conjunction with this article (freely available on the Web sites of PLoS Medicine at http://www.plosmedicine.org/, Annals of Internal Medicine at http://www.annals.org/, and Epidemiology at http://www.epidem.com/). Information on the STROBE Initiative is available at http://www.strobe-statement.org.
