# Supplementary material for: Associations between multimorbidity and adverse health outcomes in UK Biobank and the SAIL Databank: A comparison of longitudinal cohort studies
Source: PLoS Med. 2022 Mar 7;19(3):e1003931. doi: 10.1371/journal.pmed.1003931 (PMC8901063; doi:10.1371/journal.pmed.1003931)
Supplement: S1 Table — GP, General Practice. (DOCX) [file pmed.1003931.s005.docx]

# UK Biobank: Comparison of participants with linked GP data versus those without GP data available

|  | Whole cohort (n=502,533) | GP data available (n=211,597) | No GP data available (n=290,936) |
| --- | --- | --- | --- |
| Mean age (sd) | 56.5 (8.1) | 56.5 (8.1) | 56.5 (8.1) |
| Sex (%) |  |  |  |
| Male | 229,132 (45.6%) | 96,060 (45.4%) | 133,072 (45.7%) |
| Female | 273,401 (54.4%) | 115,537 (54.6%) | 157,864 (54.3%) |
| Socioeconomic status |  |  |  |
| Quintile 1 (most affluent) | 100,663 (20.1%) | 42,155 (20.0%) | 58,508 (20.1%) |
| 2 | 100,096 (19.9%) | 41,628 (19.7%) | 58,468 (20.1%) |
| 3 | 100,398 (20.0%) | 43,378 (20.5%) | 57,020 (19.6%) |
| 4 | 100,375 (20.0%) | 42,531 (20.1%) | 57,844 (19.9%) |
| Quintile 5 (most deprived) | 100,378 (20.0%) | 41,581 (19.7%) | 58,797 (20.2%) |
| Self-reported LTC count* |  |  |  |
| 0 | 172,565 (34.5%) | 71,572 (34.0%) | 100,993 (34.8%) |
| 1 | 163,680 (32.7%) | 68,987 (32.7%) | 94,693 (32.7%) |
| 2 | 95,211 (19.0%) | 40,353 (19.1%) | 54,858 (18.9%) |
| 3 | 43,113 (8.6%) | 18,702 (8.9%) | 24,411 (8.4%) |
| 4 | 16,732 (3.3%) | 7,175 (3.4%) | 9,557 (3.3%) |
| 5 | 6,056 (1.2%) | 2,580 (1.2%) | 3,476 (1.2%) |
| 6 or more | 3,331 (0.7%) | 1,428 (0.7%) | 1,903 (0.7%) |
| Note that the LTC count displayed here is based on baseline assessment centre self-report of LTCs, with conditions based on the original list of conditions used in the main analysis, adapted for UK Biobank baseline self-reported data. These definitions were not used in the main analysis as equivalent (self-reported) data are not available for SAIL. Also quintiles of Townsend scores presented here are based on the UK Biobank distribution, rather than the UK national distribution in the main text. | | | |
