# Supplementary material for: Associations between multimorbidity and adverse health outcomes in UK Biobank and the SAIL Databank: A comparison of longitudinal cohort studies
Source: PLoS Med. 2022 Mar 7;19(3):e1003931. doi: 10.1371/journal.pmed.1003931 (PMC8901063; doi:10.1371/journal.pmed.1003931)
Supplement: S2 Table — Includes confidence intervals pertaining to Fig 2. LTC, long-term condition. (DOCX) [file pmed.1003931.s006.docx]

| LTC count | Observed number of UK Biobank participants | Expected number of UK Biobank participants based on modelled relationship between age, sex and LTC count in SAIL | Expected number of UK Biobank participants based on modelled relationship between age, sex, Townsend score and LTC count in SAIL |
| --- | --- | --- | --- |
| 0 | 102504 | 61168 (61047 to 61281) | 65958 (66312 to 65605) |
| 1 | 51873 | 55265 (55212 to 55317) | 56034 (56132 to 55935) |
| 2 | 29109 | 38121 (38101 to 38142) | 35213 (35162 to 35264) |
| 3 | 14892 | 23812 (23782 to 23846) | 18807 (18726 to 18887) |
| 4 | 7144 | 14186 (14153 to 14221) | 8813 (8751 to 8874) |
| 5 | 3350 | 8237 (8208 to 8267) | 3445 (3412 to 3478) |
| 6 | 1445 | 4700 (4678 to 4724) | 1084 (1071 to 1097) |
| 7 | 550 | 2634 (2618 to 2651) | 259 (255 to 263) |
| 8 | 246 | 1450 (1439 to 1462) | 68 (67 to 69) |
| 9 | 92 | 742 (735 to 749) | 15 (14 to 15) |
| 10 | 44 | 242 (239 to 245) | 3 (3 to 3) |
| 11 | 13 | 88 (86 to 89) | 1 (1 to 1) |
| 12 | 6 | 28 (27 to 28) | 0 (0 to 0) |
| 13 | 4 | 13 (13 to 13) | 0 (0 to 0) |
| 14 | 1 | 0 (0 to 0) | 0 (0 to 0) |

**Observed and expected LTC counts in UK Biobank**
