# Supplementary material for: Associations between multimorbidity and adverse health outcomes in UK Biobank and the SAIL Databank: A comparison of longitudinal cohort studies
Source: PLoS Med. 2022 Mar 7;19(3):e1003931. doi: 10.1371/journal.pmed.1003931 (PMC8901063; doi:10.1371/journal.pmed.1003931)
Supplement: S3 Table — Observed rates of all-cause mortality, unscheduled hospitalisation, and MACE in UK Biobank and SAIL, by number of baseline LTCs. LTC, long-term condition; MACE, major adverse cardiovascular event; SAIL, Secure Anonymised Information Linkage. (DOCX) [file pmed.1003931.s007.docx]

| **Observed event rates in UK Biobank and SAIL** | | | | | | |
| --- | --- | --- | --- | --- | --- | --- |
|  | **Number of events (rate per 1000 person years)** | | | | | |
|  | **All-cause mortality** | | **Unscheduled hospitalisation** | | **MACE** | |
|  | **SAIL** | **UK Biobank** | **SAIL** | **UK Biobank** | **SAIL** | **UK Biobank** |
| **0 LTCs** | 5835 (3.4) | 1892 (2.5) | 70869 (42) | 33140 (43) | 4684 (2.6) | 1487 (1.9) |
| **1 LTC** | 5932 (4.9) | 1427 (3.7) | 76770 (64) | 20662 (54) | 4365 (3.6) | 957 (2.5) |
| **2 LTCs** | 6477 (7.1) | 1128 (5.2) | 83035 (91) | 16082 (76) | 4416 (4.8) | 669 (3.1) |
| **3 LTCs** | 5901 (10.0) | 775 (7.1) | 74908 (127) | 11477 (105) | 3726 (6.3) | 471 (4.3) |
| **4 LTCs** | 4961 (14.2) | 460 (8.8) | 61228 (175) | 7094 (136) | 2886 (8.3) | 303 (5.8) |
| **5 LTCs** | 3687 (18.9) | 288 (11.9) | 44697 (229) | 4268 (176) | 2103 (10.8) | 167 (6.8) |
| **≥6 LTCs** | 5684 (30.0) | 282 (16.6) | 68070 (359) | 4005 (235) | 3081 (16.3) | 125 (7.2) |
