# Supplementary material for: Associations between multimorbidity and adverse health outcomes in UK Biobank and the SAIL Databank: A comparison of longitudinal cohort studies
Source: PLoS Med. 2022 Mar 7;19(3):e1003931. doi: 10.1371/journal.pmed.1003931 (PMC8901063; doi:10.1371/journal.pmed.1003931)
Supplement: S4 Table — Includes unadjusted hazard ratios for those presented in the main analysis. (DOCX) [file pmed.1003931.s008.docx]

| **Unadjusted hazard ratios (from analyses for which adjusted hazard ratios are shown in main text figures)** | | |
| --- | --- | --- |
| **Individual conditions** | SAIL | UKB |
| Alcohol_related disorders | 4.52 (4.35-4.7) | 4.7 (4.09-5.4) |
| Anorexia_or_bulimia | 1.09 (0.77-1.54) | 0.66 (0.32-1.39) |
| Anxiety | 1.19 (1.16-1.22) | 1.06 (0.98-1.14) |
| Asthma | 1.87 (1.82-1.93) | 1.46 (1.32-1.62) |
| Atrial_fibrillation | 3.59 (3.42-3.77) | 3.46 (3.04-3.94) |
| Blindness_and_low_vision | 2.96 (2.71-3.24) | 1.91 (1.36-2.68) |
| Bronchiectasis | 3.41 (3.03-3.84) | 2.32 (1.78-3) |
| Cancer | 2.53 (2.46-2.61) | 2.96 (2.77-3.16) |
| Chronic_kidney_disease | 3 (2.9-3.1) | 2.68 (2.18-3.28) |
| Chronic_liver_disease | 3.99 (3.68-4.32) | 2.26 (1.76-2.91) |
| Chronic_sinusitis | 0.76 (0.7-0.83) | 0.87 (0.7-1.09) |
| COPD | 4.34 (4.22-4.46) | 3.79 (3.42-4.2) |
| Coronary Heart disease | 3.34 (3.21-3.47) | 3.04 (2.63-3.52) |
| Deafness | 1.33 (1.28-1.38) | 1.3 (1.15-1.46) |
| Dementia | 15.04 (13.72-16.49) | 14.35 (8.65-23.82) |
| Depression | 1.38 (1.35-1.41) | 1.27 (1.19-1.36) |
| Diabetes | 2.71 (2.65-2.78) | 2.9 (2.69-3.12) |
| Diverticular_disease | 1.68 (1.58-1.79) | 1.52 (1.28-1.87) |
| Epilepsy_(Currently_treated) | 2.73 (2.57-2.91) | 1.89 (1.45-2.45) |
| Glaucoma | 1.86 (1.72-2.01) | 1.86 (1.57-2.2) |
| Heart_failure | 6.71 (6.36-7.08) | 7.37 (6.06-8.95) |
| Hypertension | 2 (2-2.04) | 1.81 (1.69-1.94) |
| Inflammatory_bowel_disease | 1.33 (1.21-1.47) | 1.46 (1.11-1.94) |
| Irritable_bowel_syndrome | 0.84 (0.8-0.88) | 0.73 (0.59-0.89) |
| Learning_disability | 3.11 (2.83-3.42) | 1.64 (0.68-3.94) |
| Migraine | 0.76 (0.68-0.84) | 0.56 (0.42-0.73) |
| Multiple_sclerosis | 2.93 (2.6-3.31) | 1.82 (1.33-2.48) |
| Other_psychoactive_substance_misuse | 2.66 (2.49-2.85) | 2.19 (1.49-3.21) |
| Parkinsons_disease | 5.67 (5.03-6.38) | 4.94 (3.72-6.57) |
| Peripheral_vascular_disease | 2.98 (2.86-3.1) | 2.78 (2.48-3.11) |
| Prostate_disorders | 1.93 (1.82-2.06) | 2.84 (2.49-3.24) |
| Psoriasis_or_eczema | 1.64 (1.53-1.77) | 1.45 (1.22-1.73) |
| Rheumatoid_arthritis_Inflammatory_arthropathies_and_connective_tissue_disorders | 1.78 (1.72-1.84) | 2.22 (2.03-2.42) |
| Schizophrenia_or_bipolar_disorder | 2.76 (2.58-2.95) | 1.84 (1.42-2.39) |
| Stroke_or_TIA | 3.81 (3.65-3.97) | 3.18 (2.73-3.7) |
| Thyroid_disease | 1.17 (1.12-1.22) | 1.02 (0.88-1.18) |
| Treated_constipation | 3.09 (2.96-3.22) | 1.58 (1.44-1.74) |
| Treated_dyspepsia | 1.76 (1.71-0.18) | 2.65 (2.42-2.9) |
| Viral_hepatitis | 2.06 (1.3-3.28) | 1.34 (0.78-2.32) |
| Painful_condition | 2.51 (2.49-2.85) | 1.94 (1.83-2.06) |
| **All cause mortality** |  |  |
| 0 | ref | ref |
| 1 | 1.44 (1.39-1.5) | 1.5 (1.4-1.6) |
| 2 | 2.06 (1.99-2.14) | 2.12 (1.97-2.29) |
| 3 | 2.92 (2.81-3.02) | 2.87 (2.64-3.12) |
| 4 | 4.15 (3.99-4.31) | 3.58 (3.23-3.96) |
| 5 | 5.53 (5.31-5.77) | 4.84 (4.27-5.47) |
| 6+ | 8.78 (8.47-9.11) | 6.77 (5.97-7.67) |
| Hospitalisation (IRR) |  |  |
| 0 | ref | ref |
| 1 | 1.53 (1.52-1.55) | 1.23 (1.21-1.25) |
| 2 | 2.17 (2.15-2.19) | 1.71 (1.68-1.74) |
| 3 | 3.04 (3.01-3.07) | 2.38 (2.33-2.43) |
| 4 | 4.2 (4.16-4.25) | 3.07 (2.99-3.15) |
| 5 | 5.5 (5.43-5.56) | 3.94 (3.82-4.07) |
| 6+ | 8.62 (8.53-8.71) | 5.16 (5-5.33) |
| MACE |  |  |
| 0 | ref | ref |
| 1 | 1.32 (1.27-1.38) | 1.27 (1.18-1.38) |
| 2 | 1.75 (1.68-1.83) | 1.6 (1.46-1.75) |
| 3 | 2.31 (2.21-2.41) | 2.21 (1.99-2.45) |
| 4 | 3.02 (2.89-3.17) | 2.98 (2.63-3.37) |
| 5 | 3.96 (3.76-4.17) | 3.53 (3.01-4.14) |
| 6+ | 6.02 (5.75-6.3) | 3.71 (3.09-4.45) |
