# Supplementary material for: Associations between multimorbidity and adverse health outcomes in UK Biobank and the SAIL Databank: A comparison of longitudinal cohort studies
Source: PLoS Med. 2022 Mar 7;19(3):e1003931. doi: 10.1371/journal.pmed.1003931 (PMC8901063; doi:10.1371/journal.pmed.1003931)
Supplement: S5 Table — Descriptive statistics of Cambridge multimorbidity score in UK Biobank and SAIL. SAIL, Secure Anonymised Information Linkage. (DOCX) [file pmed.1003931.s009.docx]

# Cambridge multimorbidity score: Comparison of UK Biobank and SAIL

## Distributions of General Weight Scores

| Cambridge score | UK Biobank (n=211,597) | SAIL (n=852,291) |
| --- | --- | --- |
| 0-1 | 138,865 (65.6%) | 459,609 (53.9%) |
| 1.1-2 | 40,935 (19.3%) | 217,203 (25.5%) |
| 2.1-3 | 23,914 (11.3%) | 116,314 (13.6%) |
| 3.1-4 | 5,824 (2.8%) | 39,491 (4.6%) |
| 4.1-5 | 1,588 (0.8%) | 13,720 (1.6%) |
| 5.1-6 | 351 (0.2%) | 4,257 (0.5%) |
| 6.1-7 | 96 (0.04%) | 1,282 (0.2%) |
| 7.1-8 | 19 (0.009%) | 310 (0.04%) |
| 8.1-9 | 5 (0.002%) | 75 (0.009%) |
| >9 | 0 | 30 (0.003%) |
