# Supplementary material for: Associations between multimorbidity and adverse health outcomes in UK Biobank and the SAIL Databank: A comparison of longitudinal cohort studies
Source: PLoS Med. 2022 Mar 7;19(3):e1003931. doi: 10.1371/journal.pmed.1003931 (PMC8901063; doi:10.1371/journal.pmed.1003931)
Supplement: S1 Fig — Observed and expected LTC counts in SAIL, stratified by age, sex, and socioeconomic status. LTC, long-term condition; SAIL, Secure Anonymised Information Linkage. (PDF) [file pmed.1003931.s010.pdf]

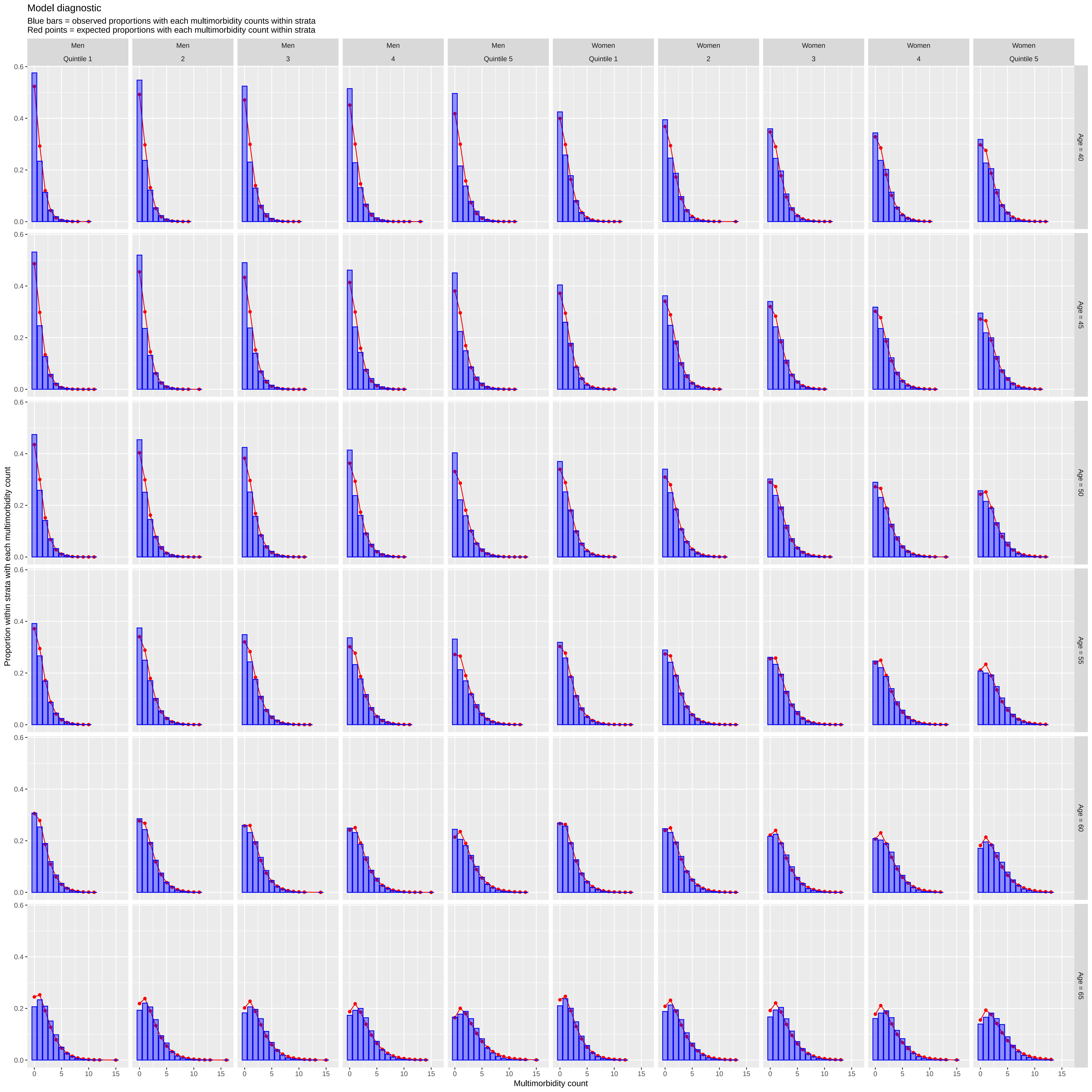
Model diagnostic
Blue bars = observed proportions with each multimorbidity counts within strata
Red points = expected proportions with each multimorbidity count within strata
Men Quintile 1
Men 2
Men 3
Men 4
Men Quintile 5
Women Quintile 1
Women 2
Women 3
Women 4
Women Quintile 5
Age = 40
Age = 45
Age = 50
Age = 55
Age = 60
Age = 65
Proportion within strata with each multimorbidity count
Multimorbidity count
0.0
0.2
0.4
0.6
0
5
10
15
