# Supplementary material for: Associations between multimorbidity and adverse health outcomes in UK Biobank and the SAIL Databank: A comparison of longitudinal cohort studies
Source: PLoS Med. 2022 Mar 7;19(3):e1003931. doi: 10.1371/journal.pmed.1003931 (PMC8901063; doi:10.1371/journal.pmed.1003931)
Supplement: S2 Fig — Line indicates the modelled values for each cohort (stratified by age), shaded area indicates 95% CIs, points indicate the observed proportion of deaths within each stratum of age and LTC count. Size of the point indicates the number of events per strata. CI, confidence interval; LTC, long-term condition; SAIL, Secure Anonymised Information Linkage. (PDF) [file pmed.1003931.s011.pdf]

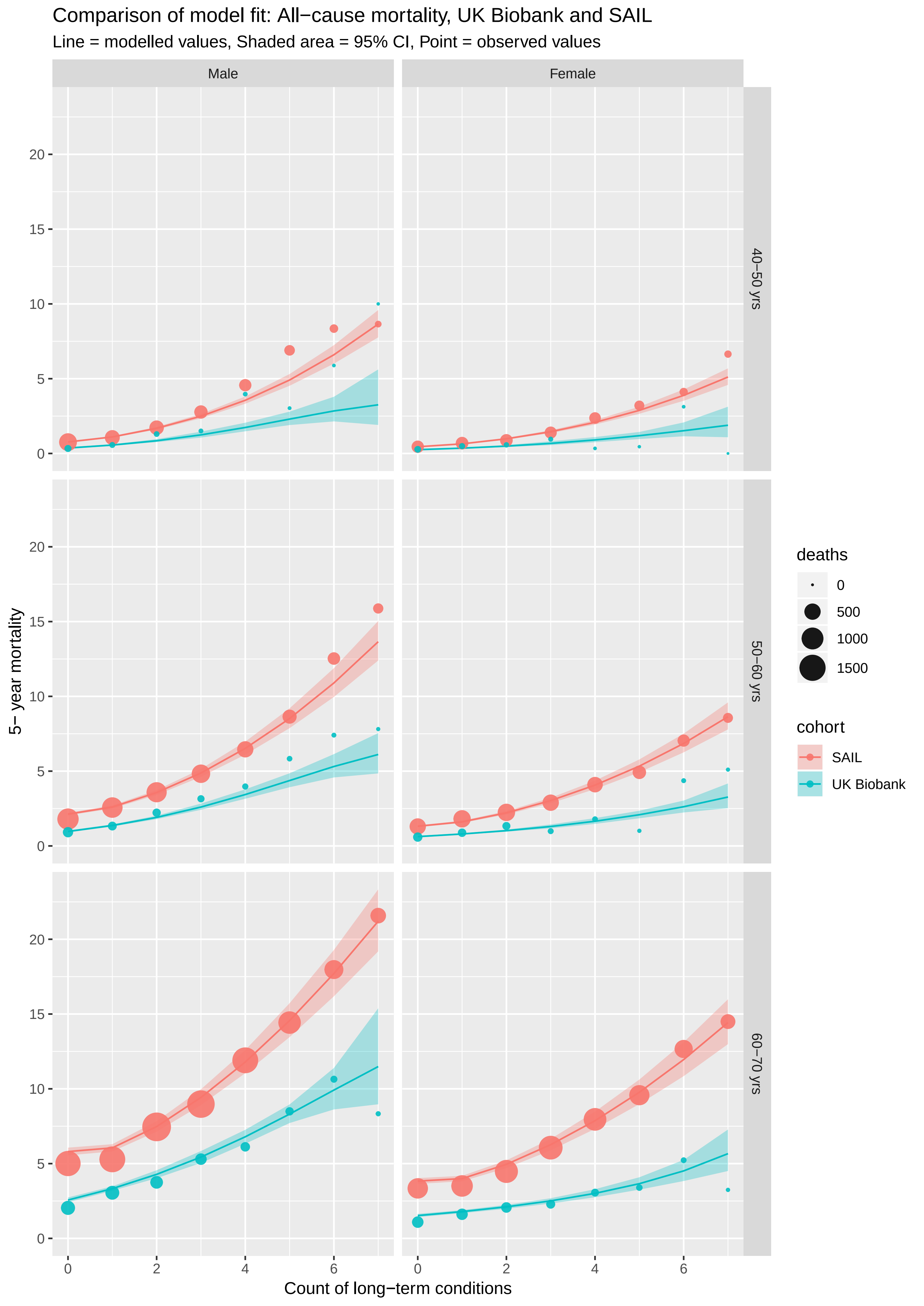

Comparison of model fit: All−cause mortality, UK Biobank and SAIL
Line = modelled values, Shaded area = 95% CI, Point = observed values
Male
Female
40−50 yrs
50−60 yrs
60−70 yrs
5− year mortality
Count of long−term conditions
deaths
0
500
1000
1500
cohort
SAIL
UK Biobank
