# Supplementary material for: Associations between multimorbidity and adverse health outcomes in UK Biobank and the SAIL Databank: A comparison of longitudinal cohort studies
Source: PLoS Med. 2022 Mar 7;19(3):e1003931. doi: 10.1371/journal.pmed.1003931 (PMC8901063; doi:10.1371/journal.pmed.1003931)

Model fit: Negative binomial model for unscheduled hospitalisation events in SAIL. Observed number of hospitalisations (bars) and expected (red points). Counts <5 are redacted.

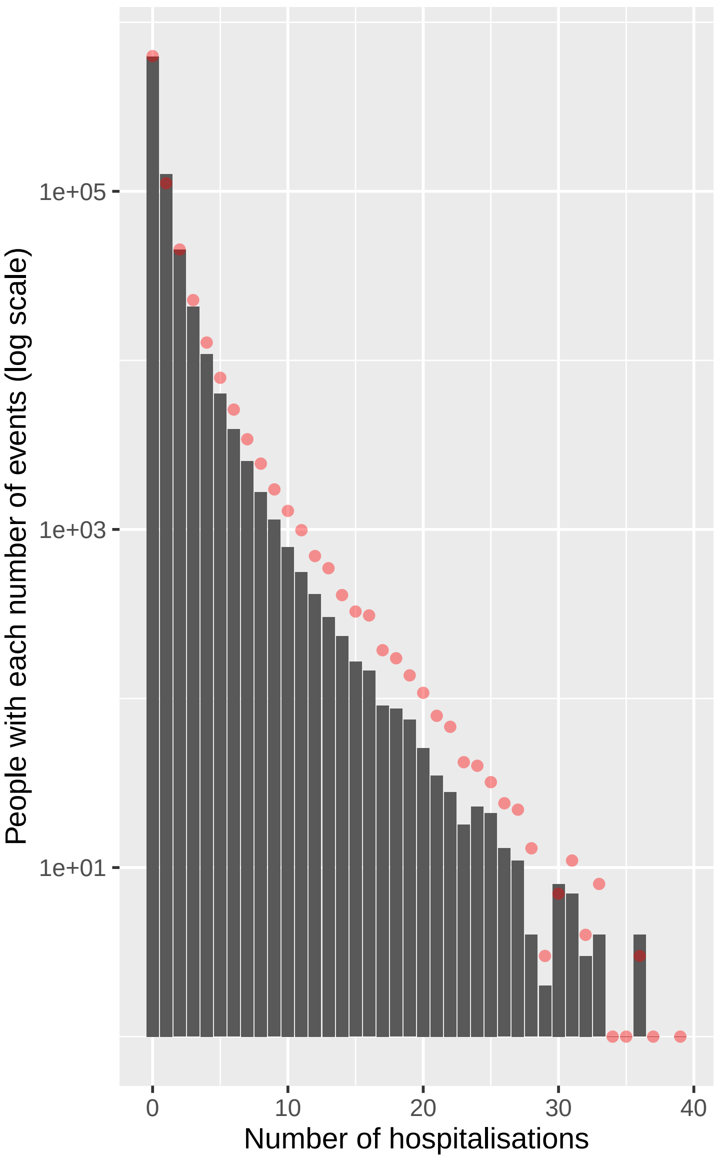

Supplement: S4 Fig — Observed and expected number of unscheduled hospitalisations. SAIL, Secure Anonymised Information Linkage. (PDF) [file pmed.1003931.s013.pdf]
