# Supplementary material for: Associations between multimorbidity and adverse health outcomes in UK Biobank and the SAIL Databank: A comparison of longitudinal cohort studies
Source: PLoS Med. 2022 Mar 7;19(3):e1003931. doi: 10.1371/journal.pmed.1003931 (PMC8901063; doi:10.1371/journal.pmed.1003931)

Unscheduled hospitalisations (predicted at baseline age 60 years, Townsend score at SAIL mean) – using hospitalization weights

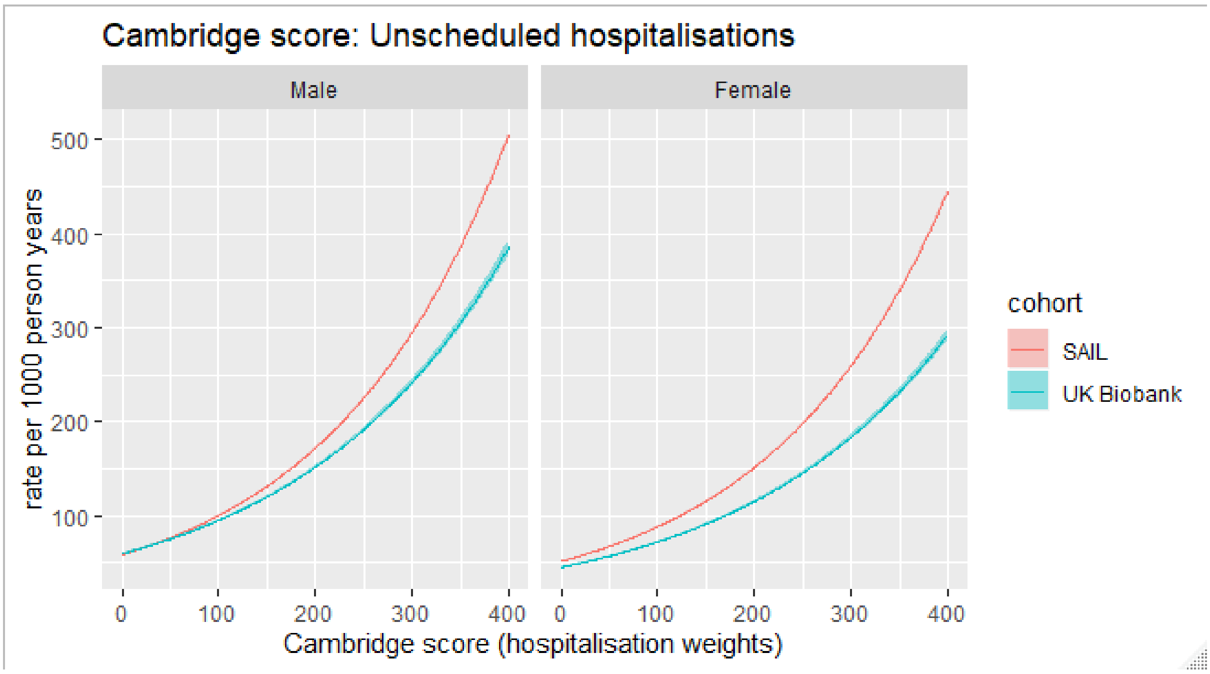

Supplement: S9 Fig — Line indicates the modelled values for each cohort; shaded area indicates 95% CIs. CI, confidence interval; SAIL, Secure Anonymised Information Linkage. (PDF) [file pmed.1003931.s018.pdf]
