# Supplementary figures and images for: Associations between multimorbidity and adverse health outcomes in UK Biobank and the SAIL Databank: A comparison of longitudinal cohort studies
Source: PLoS Med. 2022 Mar 7;19(3):e1003931. doi: 10.1371/journal.pmed.1003931 (PMC8901063; doi:10.1371/journal.pmed.1003931)

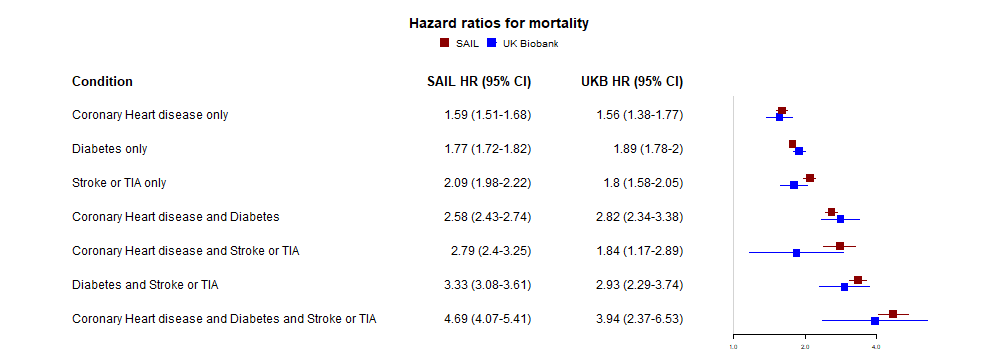

Supplement: S11 Fig — CI, confidence interval; HR, hazard ratio; SAIL, Secure Anonymised Information Linkage; TIA, transient ischaemic attack; UKB, UK Biobank. (PNG) [file pmed.1003931.s020.png]

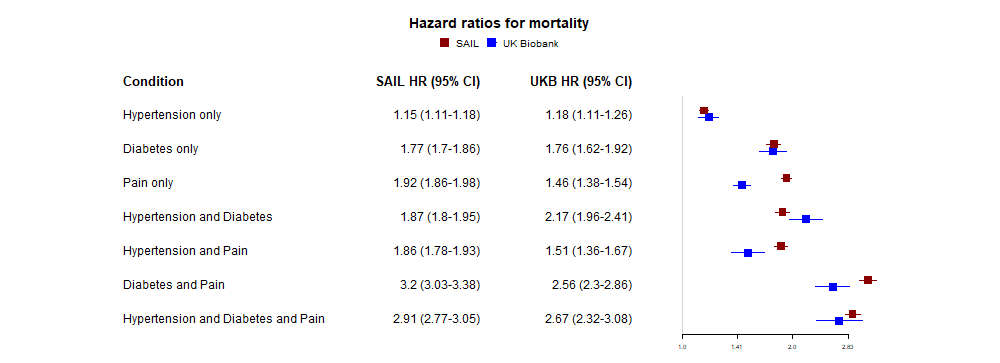

Supplement: S12 Fig — CI, confidence interval; HR, hazard ratio; SAIL, Secure Anonymised Information Linkage; UKB, UK Biobank. (PNG) [file pmed.1003931.s021.png]

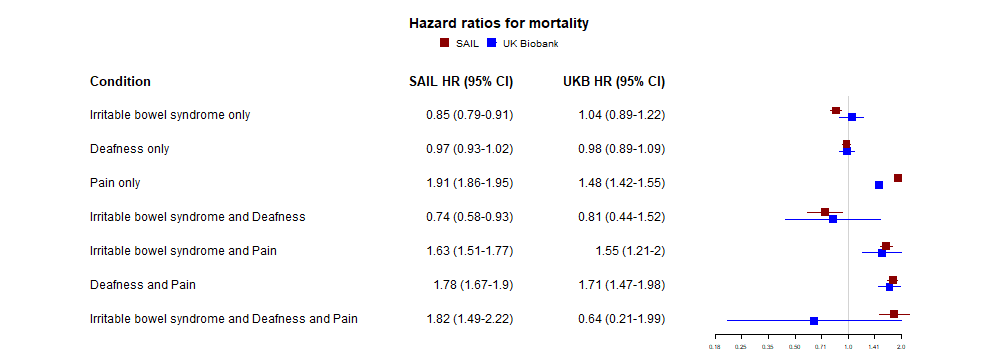

Supplement: S13 Fig — CI, confidence interval; HR, hazard ratio; SAIL, Secure Anonymised Information Linkage; UKB, UK Biobank. (PNG) [file pmed.1003931.s022.png]

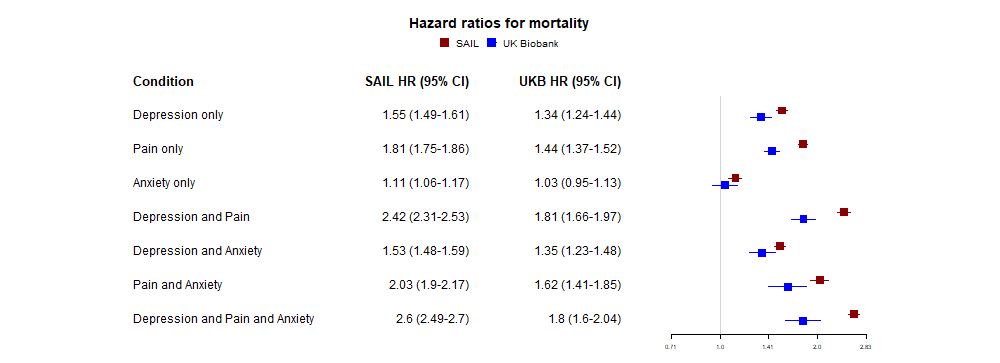

Supplement: S14 Fig — CI, confidence interval; HR, hazard ratio; SAIL, Secure Anonymised Information Linkage; UKB, UK Biobank. (PNG) [file pmed.1003931.s023.png]

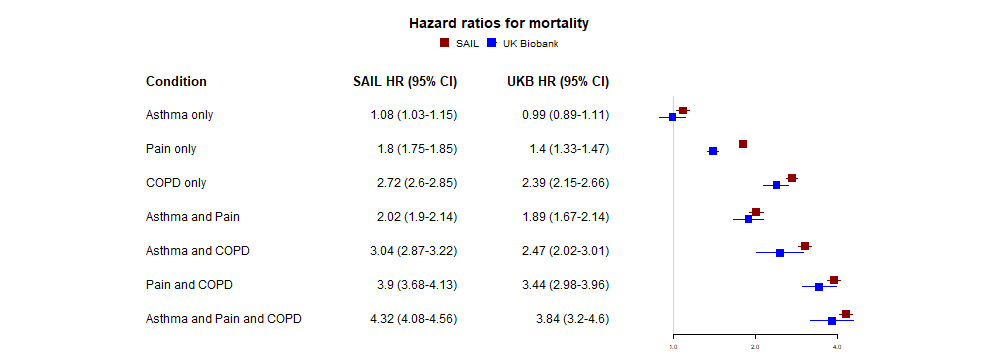

Supplement: S15 Fig — CI, confidence interval; COPD, chronic obstructive pulmonary disease; HR, hazard ratio; SAIL, Secure Anonymised Information Linkage; UKB, UK Biobank. (PNG) [file pmed.1003931.s024.png]

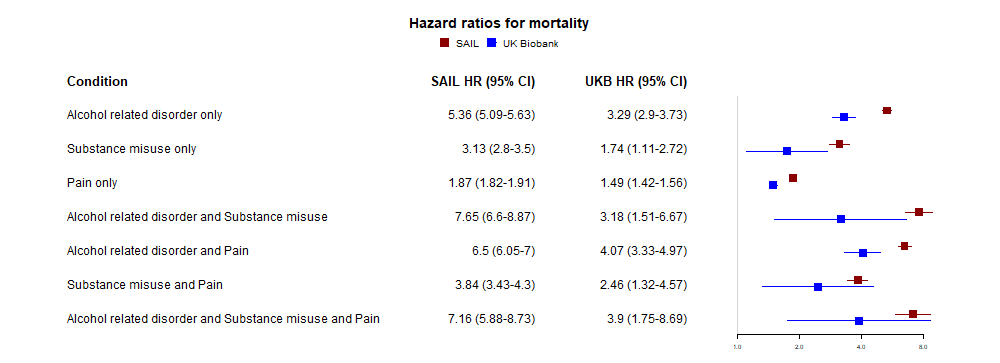

Supplement: S16 Fig — CI, confidence interval; HR, hazard ratio; SAIL, Secure Anonymised Information Linkage; UKB, UK Biobank. (PNG) [file pmed.1003931.s025.png]

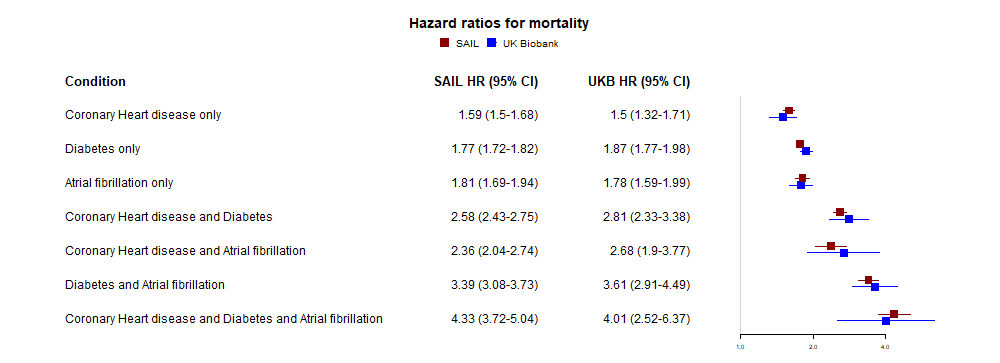

Supplement: S17 Fig — CI, confidence interval; HR, hazard ratio; SAIL, Secure Anonymised Information Linkage; UKB, UK Biobank. (PNG) [file pmed.1003931.s026.png]

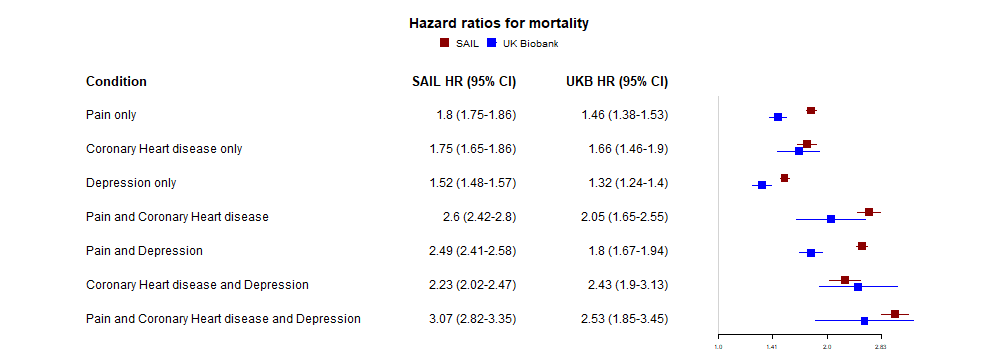

Supplement: S18 Fig — CI, confidence interval; HR, hazard ratio; SAIL, Secure Anonymised Information Linkage; UKB, UK Biobank. (PNG) [file pmed.1003931.s027.png]

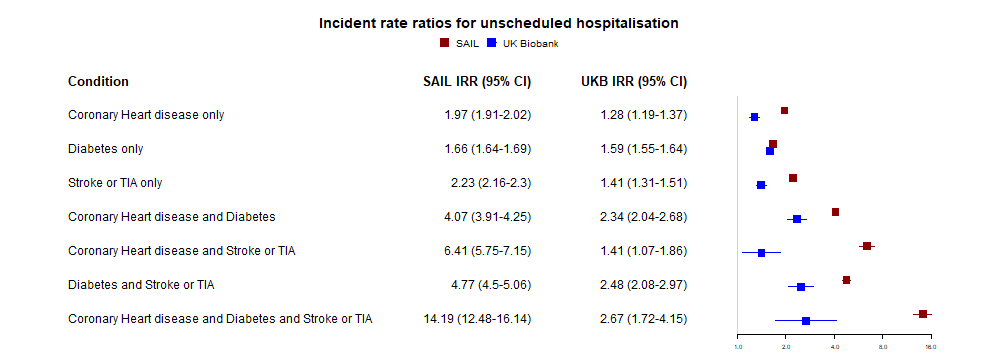

Supplement: S19 Fig — CI, confidence interval; IRR, incidence rate ratio; SAIL, Secure Anonymised Information Linkage; TIA, transient ischaemic attack; UKB, UK Biobank. (PNG) [file pmed.1003931.s028.png]

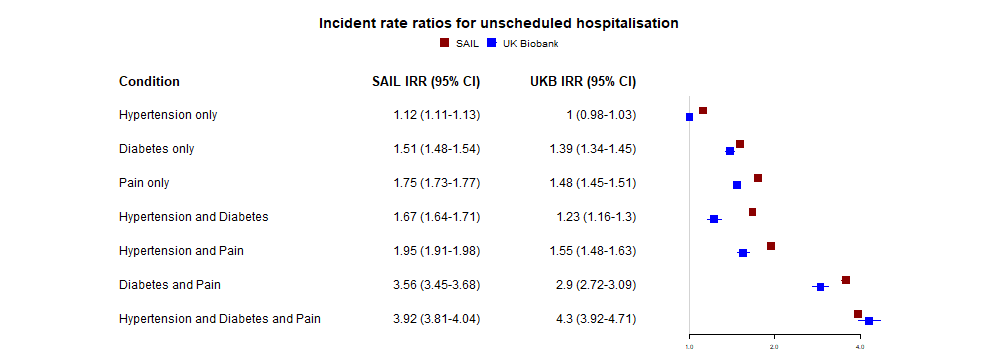

Supplement: S20 Fig — CI, confidence interval; IRR, incidence rate ratio; SAIL, Secure Anonymised Information Linkage; UKB, UK Biobank. (PNG) [file pmed.1003931.s029.png]

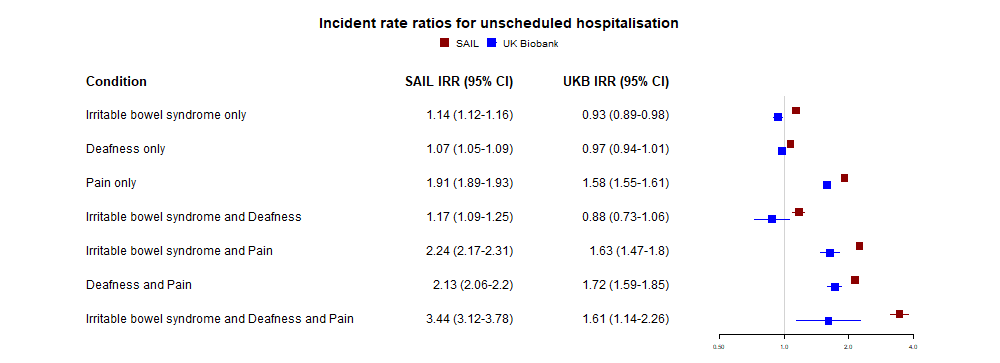

Supplement: S21 Fig — CI, confidence interval; IRR, incidence rate ratio; SAIL, Secure Anonymised Information Linkage; UKB, UK Biobank. (PNG) [file pmed.1003931.s030.png]

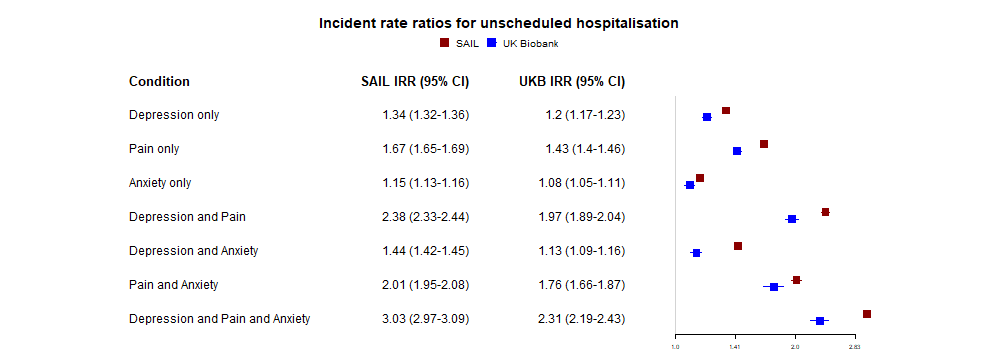

Supplement: S22 Fig — CI, confidence interval; IRR, incidence rate ratio; SAIL, Secure Anonymised Information Linkage; UKB, UK Biobank. (PNG) [file pmed.1003931.s031.png]

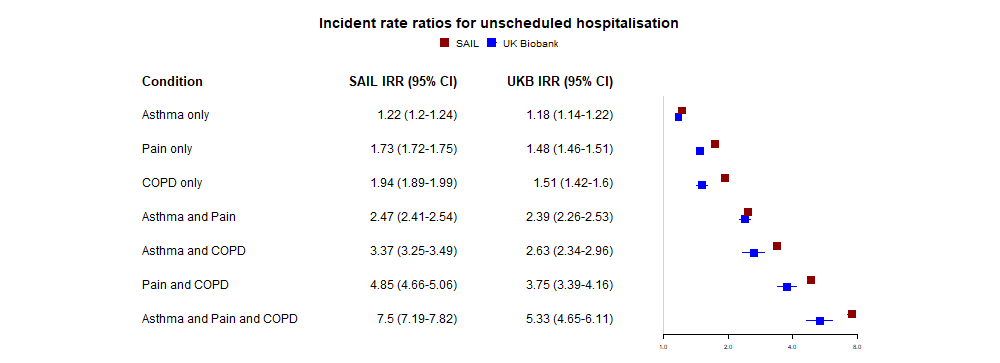

Supplement: S23 Fig — CI, confidence interval; COPD, chronic obstructive pulmonary disease; IRR, incidence rate ratio; SAIL, Secure Anonymised Information Linkage; UKB, UK Biobank. (PNG) [file pmed.1003931.s032.png]

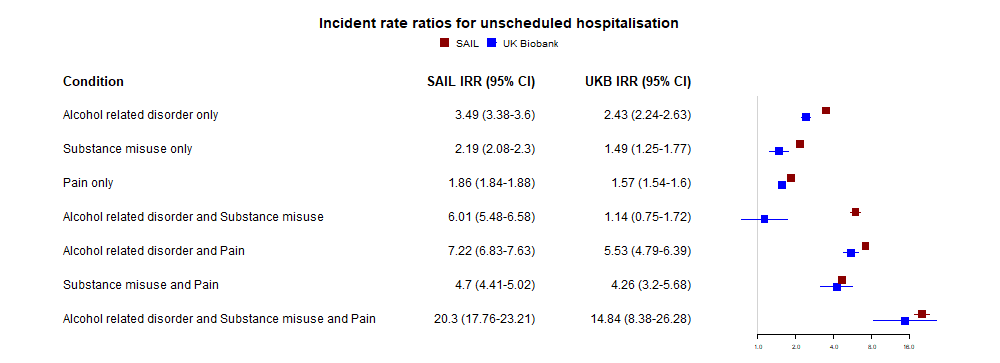

Supplement: S24 Fig — CI, confidence interval; IRR, incidence rate ratio; SAIL, Secure Anonymised Information Linkage; UKB, UK Biobank. (PNG) [file pmed.1003931.s033.png]

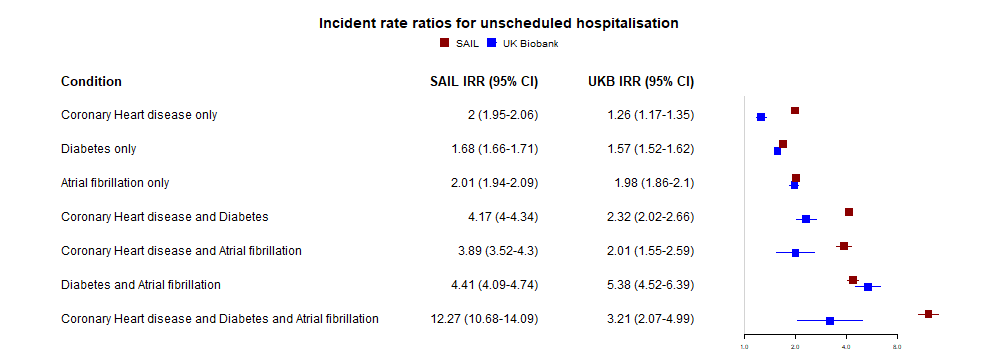

Supplement: S25 Fig — CI, confidence interval; IRR, incidence rate ratio; SAIL, Secure Anonymised Information Linkage; UKB, UK Biobank. (PNG) [file pmed.1003931.s034.png]

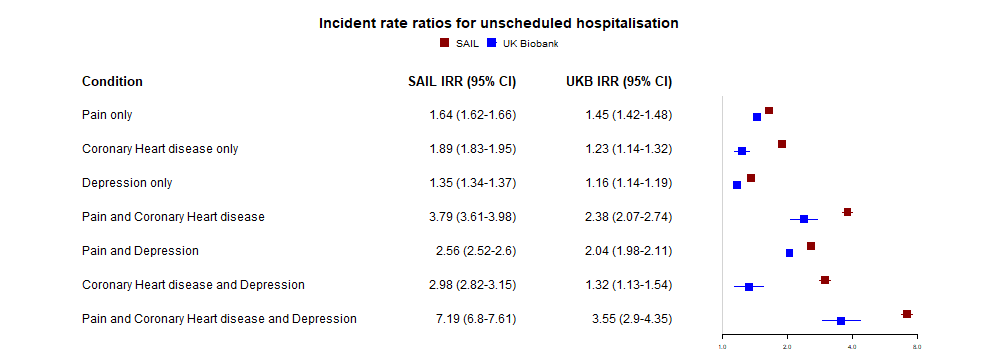

Supplement: S26 Fig — CI, confidence interval; IRR, incidence rate ratio; SAIL, Secure Anonymised Information Linkage; UKB, UK Biobank. (PNG) [file pmed.1003931.s035.png]

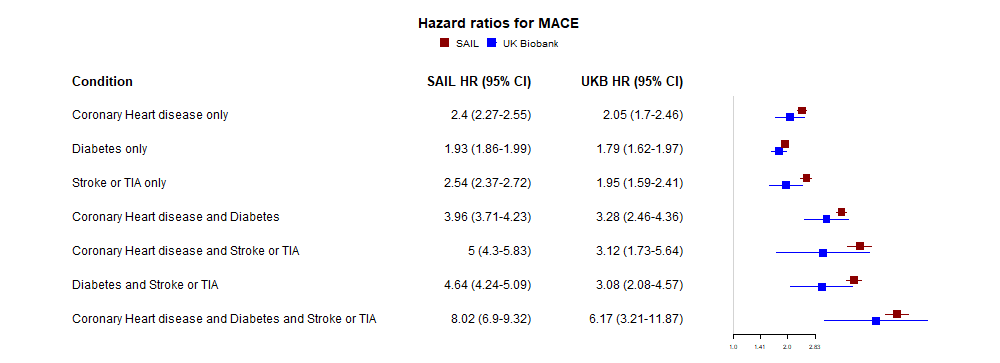

Supplement: S27 Fig — CI, confidence interval; HR, hazard ratio; MACE, major adverse cardiovascular event; SAIL, Secure Anonymised Information Linkage; TIA, transient ischaemic attack; UKB, UK Biobank. (PNG) [file pmed.1003931.s036.png]

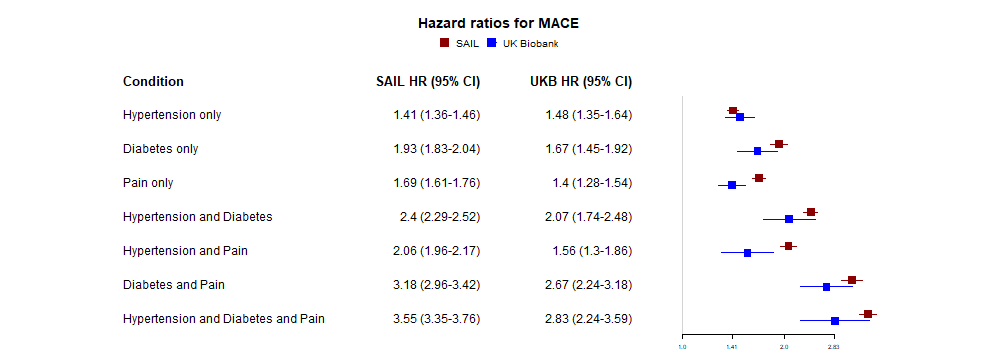

Supplement: S28 Fig — CI, confidence interval; HR, hazard ratio; MACE, major adverse cardiovascular event; SAIL, Secure Anonymised Information Linkage; UKB, UK Biobank. (PNG) [file pmed.1003931.s037.png]

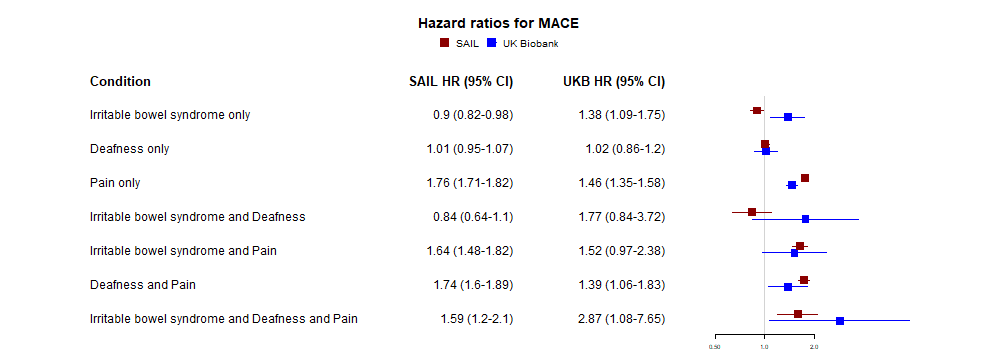

Supplement: S29 Fig — CI, confidence interval; HR, hazard ratio; MACE, major adverse cardiovascular event; SAIL, Secure Anonymised Information Linkage; UKB, UK Biobank. (PNG) [file pmed.1003931.s038.png]

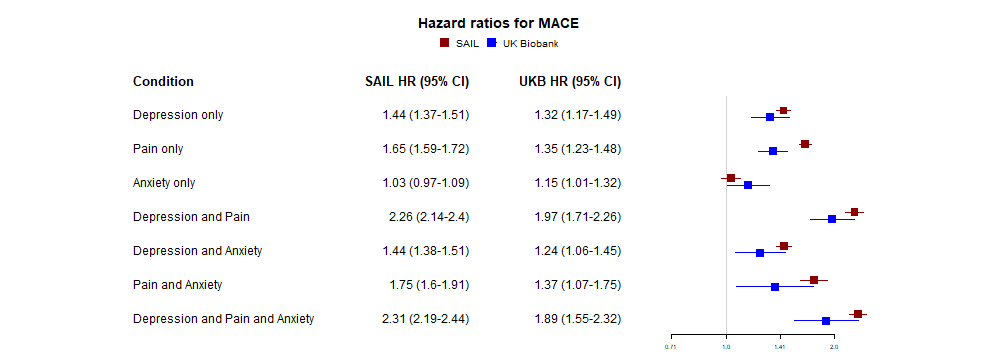

Supplement: S30 Fig — CI, confidence interval; HR, hazard ratio; MACE, major adverse cardiovascular event; SAIL, Secure Anonymised Information Linkage; UKB, UK Biobank. (PNG) [file pmed.1003931.s039.png]

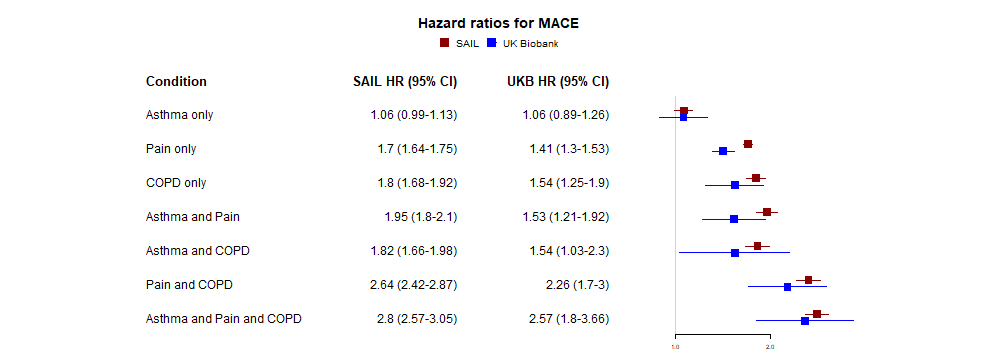

Supplement: S31 Fig — CI, confidence interval; COPD, chronic obstructive pulmonary disease; HR, hazard ratio; MACE, major adverse cardiovascular event; SAIL, Secure Anonymised Information Linkage; UKB, UK Biobank. (PNG) [file pmed.1003931.s040.png]

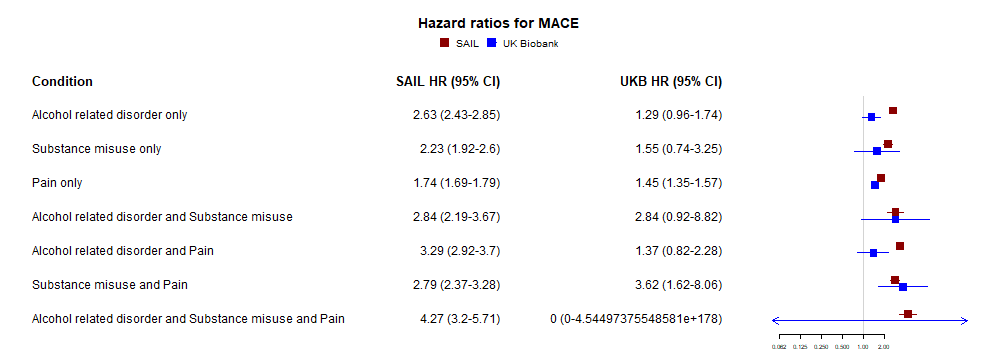

Supplement: S32 Fig — CI, confidence interval; HR, hazard ratio; MACE, major adverse cardiovascular event; SAIL, Secure Anonymised Information Linkage; UKB, UK Biobank. (PNG) [file pmed.1003931.s041.png]

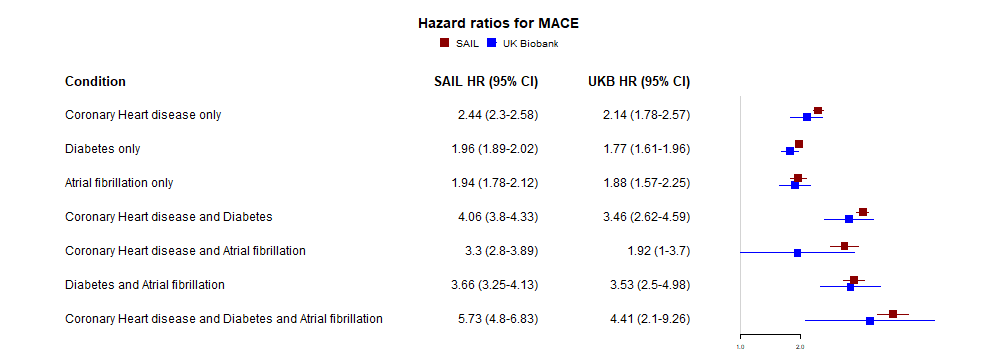

Supplement: S33 Fig — CI, confidence interval; HR, hazard ratio; MACE, major adverse cardiovascular event; SAIL, Secure Anonymised Information Linkage; UKB, UK Biobank. (PNG) [file pmed.1003931.s042.png]

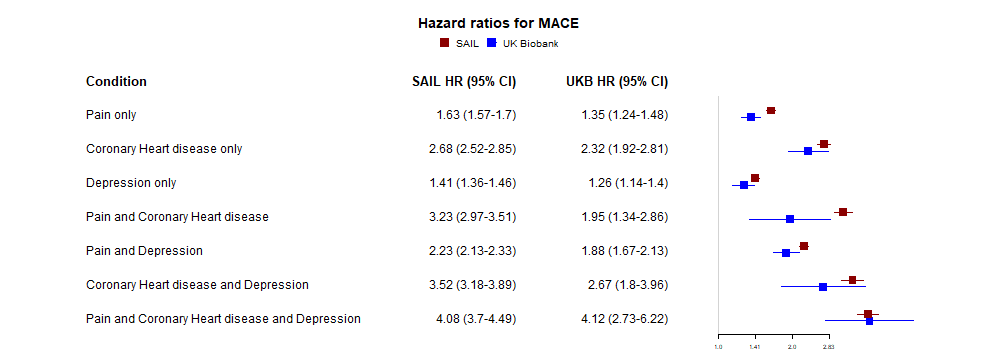

Supplement: S34 Fig — CI, confidence interval; HR, hazard ratio; MACE, major adverse cardiovascular event; SAIL, Secure Anonymised Information Linkage; UKB, UK Biobank. (PNG) [file pmed.1003931.s043.png]
