# Supplementary material for: Associations between multimorbidity and adverse health outcomes in UK Biobank and the SAIL Databank: A comparison of longitudinal cohort studies
Source: PLoS Med. 2022 Mar 7;19(3):e1003931. doi: 10.1371/journal.pmed.1003931 (PMC8901063; doi:10.1371/journal.pmed.1003931)
Supplement: S35 Fig — Comparison of full cohort and area-specific estimates. LTC, long-term condition; SAIL, Secure Anonymised Information Linkage. (PDF) [file pmed.1003931.s044.pdf]

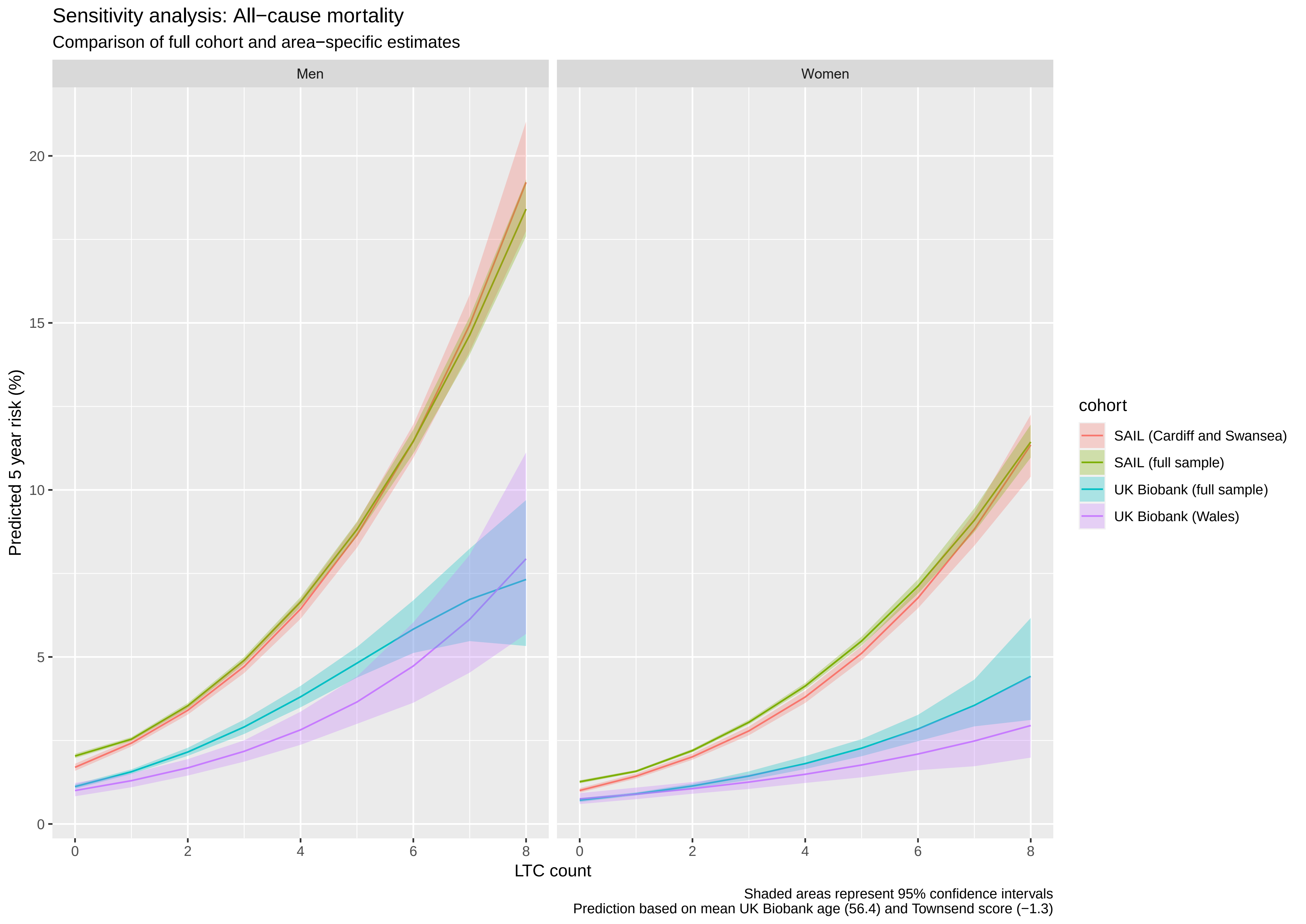

Sensitivity analysis: All−cause mortality
Comparison of full cohort and area−specific estimates
Men
Women
Predicted 5 year risk (%)
0
5
10
15
20
0
2
4
6
8
LTC count
cohort
SAIL (Cardiff and Swansea)
SAIL (full sample)
UK Biobank (full sample)
UK Biobank (Wales)
Shaded areas represent 95% confidence intervals
Prediction based on mean UK Biobank age (56.4) and Townsend score (−1.3)
