# Supplementary material for: Associations between multimorbidity and adverse health outcomes in UK Biobank and the SAIL Databank: A comparison of longitudinal cohort studies
Source: PLoS Med. 2022 Mar 7;19(3):e1003931. doi: 10.1371/journal.pmed.1003931 (PMC8901063; doi:10.1371/journal.pmed.1003931)
Supplement: S36 Fig — Comparison of full cohort and area-specific estimates. LTC, long-term condition; SAIL, Secure Anonymised Information Linkage. (PDF) [file pmed.1003931.s045.pdf]

Sensitivity analysis: Unscheduled hospitalisation

Comparison of full cohort and area−specific estimates

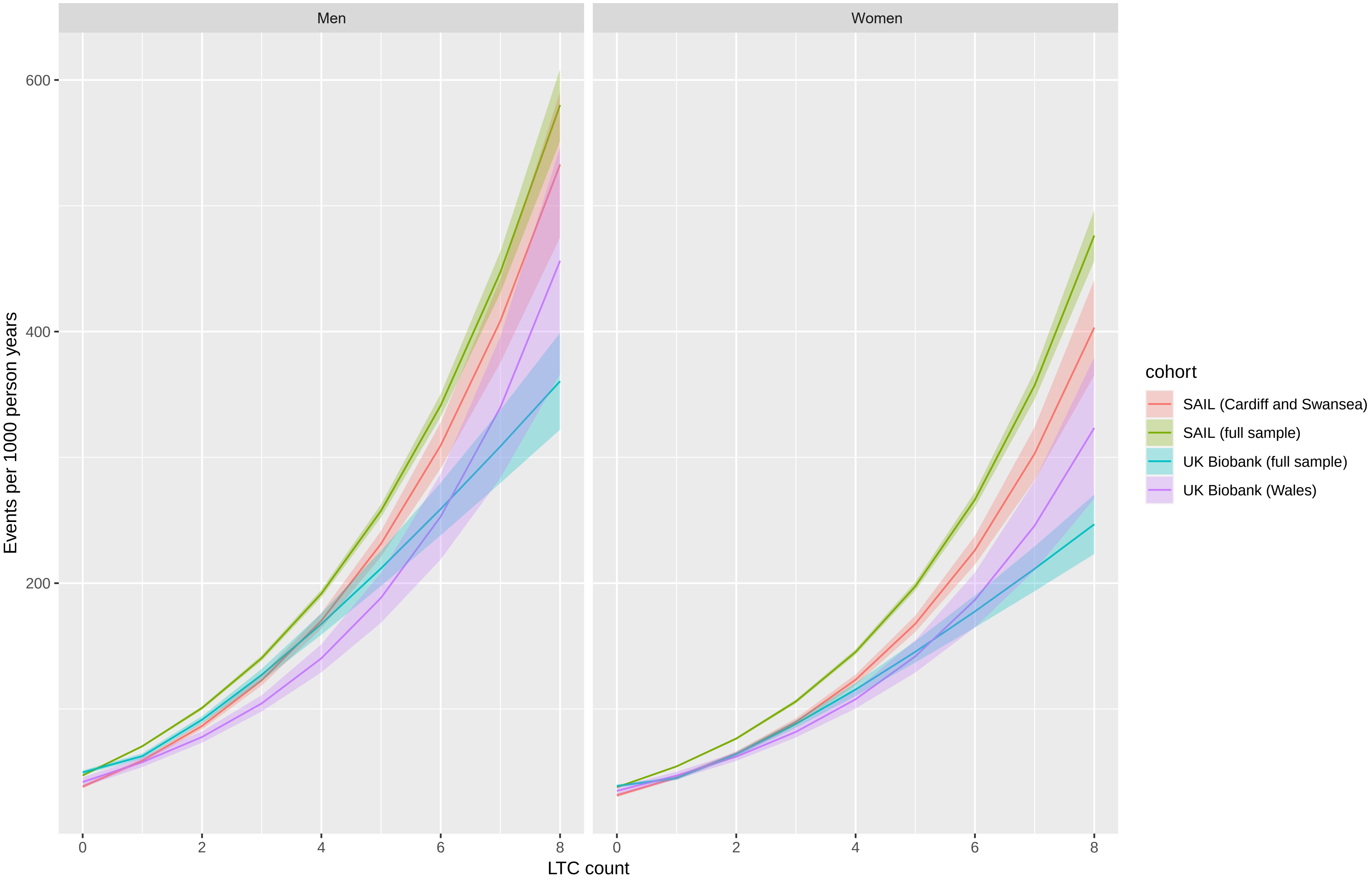

Shaded areas represent 95% confidence intervals
Prediction based on mean UK Biobank age (56.4) and Townsend score (−1.3)
