# Supplementary material for: Associations between multimorbidity and adverse health outcomes in UK Biobank and the SAIL Databank: A comparison of longitudinal cohort studies
Source: PLoS Med. 2022 Mar 7;19(3):e1003931. doi: 10.1371/journal.pmed.1003931 (PMC8901063; doi:10.1371/journal.pmed.1003931)
Supplement: Attachment — Submitted filename: PLOS Medicine response to reviewers submitted.docx [file pmed.1003931.s047.docx]

Dear Editors,

**Re: Associations between multimorbidity and adverse health outcomes in UK Biobank and the SAIL Databank: A comparison of longitudinal cohort studies**

Thank you for the opportunity to revise our manuscript.

This document contains our responses to each of the editorial and reviewer comments. Original comments are in black text. Responses are in blue text. We have quoted all changes to the manuscript text with page and line references. These refer to the clean (not tracked changes) version of the resubmitted manuscript.

Thank you for considering our manuscript and we look forward to your decision in due course.

Yours sincerely

Dr Peter Hanlon

Clinical Research Fellow, Institute for Health and Wellbeing, University of Glasgow

**Editor’s Comments**

Title: Please revise your title according to PLOS Medicine's style, placing the study design in the subtitle (ie, after a colon). We suggest “Associations between multimorbidity and adverse health outcomes in UK Biobank and the SAIL Databank: A comparison of longitudinal cohort studies” or similar

We have changed the title as suggested

Abstract:
Abstract Methods and Findings:
Please include the study design and years during which data was collected for the UKBB / SAIL cohorts

We have added these details (quoted text in response to comment below).

Please provide brief demographic details of the study population (e.g. sex, age, ethnicity, etc) in each of the databanks studied.

We have added these details to the abstract. Unfortunately, data on ethnicity were not available for SAIL so are not reported in the abstract.

“These are observational analyses of cohorts identified from linked routine healthcare data from UK Biobank participants (n=211,597, age 40-70, mean age 56.5 years, 54.6% women, baseline assessment 2006-2010) and from the Secure Anonymised Information Linkage (SAIL) databank (n=852,055, age 40-70, mean age 54.2, 50.0% women, baseline January 2011).”

Page 2, lines 31-34

Please quantify the results presented in your abstract (with 95% CIs), ensuring that all numbers presented in the abstract are identical to numbers presented in the main manuscript text.

“Multimorbidity was less common in UK Biobank than SAIL (26.9% and 33.0% with ≥2 LTCs in UK Biobank and SAIL, respectively).” Page 2 lines 40-41

And

“The effect of increasing multimorbidity count on mortality, hospitalisation, and MACE was similar between both datasets at LTC counts of ≤3, however above this level UK Biobank underestimated the risk associated with multimorbidity (e.g. mortality hazard ratio for 2 LTCs 1.62 (95% CI 1.57-1.68) in SAIL and 1.51 (1.43-1.59) in UK Biobank, hazard ratio for 5 LTCs was 3.46 (3.31-3.61) in SAIL and 2.88 (2.63-3.15) in UK Biobank).” Page 2 lines 42-46

In the last sentence of the Abstract Methods and Findings section, please describe 2-3 of  the main limitations of the study's methodology.

We have added the following:

“The main limitations are that SAIL databank represents only part of the UK (Wales only) and that we lacked any data on severity of the LTCs included.” Page 3 lines 53-54

Abstract Conclusions:
Please begin your Abstract Conclusions with "In this study, we observed ..." or similar, to summarize the main findings from your study, without overstating your conclusions. Please emphasize what is new and address the implications of your study, being careful to avoid assertions of primacy. Please be more specific as to which outcomes you are referring to.

“In this study, we observed that UK Biobank accurately estimates risk of mortality, unscheduled hospitalisation, and MACE associated with LTC counts ≤3. However, for counts ≥4 estimates of magnitude of association from UK Biobank are likely to be conservative. Researchers should be mindful of these limitations of UK Biobank when conducting and interpreting analyses of multimorbidity. Nonetheless, the richness of data available in UK Biobank does offers opportunities to better understand multimorbidity, particularly where complementary data sources less susceptible to selection bias can be used to inform and qualify analyses of UK Biobank.” Page 3 lines 56-62

Author Summary:
At this stage, we ask that you include a short, non-technical Author Summary of your research to make findings accessible to a wide audience that includes both scientists and non-scientists. The Author Summary should immediately follow the Abstract in your revised manuscript. This text is subject to editorial change and should be distinct from the scientific abstract. Please see our author guidelines for more information: <https://journals.plos.org/plosmedicine/s/revising-your-manuscript#loc-author-summary>
In the final bullet point of ‘What Do These Findings Mean?’, please describe the main limitations of the study in non-technical  language.

We have added the following:

“Author Summary

## Why was this study done?

- Multimorbidity, the presence of multiple long-term conditions (LTCs), is associated with a range of adverse health outcomes.
- The UK Biobank cohort study has gathered and linked genetic, physical and clinical information on a population scale providing unique opportunities to study the impact of multimorbidity.
- However, participants in UK Biobank appear on average to be healthier than the general population (‘healthy volunteer bias’) and it is not clear if this selection bias affects estimates of the impact of multimorbidity using UK Biobank.

## What did the researchers do and find?

- We compared the prevalence of multimorbidity, and the impact of multimorbidity on adverse health outcomes, in UK Biobank and in a representative sample of people from Wales, UK (SAIL databank).
- While multimorbidity was less common in UK Biobank, the relationship between number of LTCs and mortality, hospital admissions, and major adverse cardiovascular events was similar between UK Biobank and SAIL at lower levels of multimorbidity (e.g. 2 or 3 LTCs) and for many common LTCs (e.g. hypertension, coronary artery disease, and chronic obstructive pulmonary disease).
- However, for people with higher LTC counts (e.g. 4 or more), or with for specific LTCs such as mental health conditions, UK Biobank underestimates the risk of mortality, hospitalisation and Major Adverse Cardiovascular Events.

## What do these findings mean?

- The wide range of measures gathered by UK Biobank make it a valuable resource for studying multimorbidity, and our study suggests that analyses of modest levels of multimorbidity (such as people with 2 or 3 LTCs) are likely to yield reliable estimates.
- However, for people with a higher number of LTCs or with LTCs such as mental health conditions, alcohol problems or addiction, estimates based on UK Biobank data are likely to be conservative compared to a representative sample.
- Ideally, future LTC and multimorbidity research should combine insights from both representative routine data and information rich research cohorts such as UK Biobank.
- These analyses are limited by a lack of data on the severity of LTCs.”

Pages 4-5

Introduction:
Please explain the potential importance of your study and indicate whether your study is novel, being careful to temper assertions of primacy.

We have added the following text before the study aims:

“[This study seeks to compare the relationship between multimorbidity and adverse health outcomes in UK Biobank with a representative, unselected population identified from routine health records]. This is important as it addresses the question of whether UK Biobank analyses can produce reliable estimates of the associations between multimorbidity and adverse health outcomes. Understanding and quantifying the reliability of UK Biobank in this context is needed if the strengths mentioned above are to be harnessed to meet the rising challenge of multimorbidity.” Page 7 lines 136-142

Methods:
Did your study have a prospective protocol or analysis plan? Please state this (either way) early in the Methods section. If a prospective analysis plan (from your funding proposal, IRB or other ethics committee submission, study protocol, or other planning document written before analyzing the data) was used in designing the study, please include the relevant prospectively written document with your revised manuscript as a Supporting Information file to be published alongside your study, and cite it in the Methods section. A legend for this file should be included at the end of your manuscript. If no such document exists, please make sure that the Methods section transparently describes when analyses were planned, and if/when reported analyses differed from those that were planned. Changes in the analysis-- including those made in response to peer review comments-- should be identified as such in the Methods section of the paper, with rationale. If a reported analysis was
performed based on an interesting but unanticipated pattern in the data, please be clear that the analysis was data-driven.

We have included our pre-specified analysis plan as an additional file. We have also indicated in the revisions to the methods section where any additional analyses were conducted in response to comments following peer review.

Please ensure that the study is reported according to the STROBE guideline, and include the completed STROBE checklist as Supporting Information.  Please add the following statement, or similar, to the Methods: "This study is reported as per the Strengthening the Reporting of Observational Studies in Epidemiology (STROBE) guideline (S1 Checklist)." The STROBE guideline can be found here: <http://www.equator-network.org/reporting-guidelines/strobe/> When completing the checklist, please use section and paragraph numbers, rather than page numbers which will likely no longer correspond to the appropriate sections after copy-editing.

We have added the completed STROBE checklist as an additional file and have added the requested text to the methods section.

Please provide an ethics statement in your Methods section.

We have added the following statement:

“The UK Biobank has full ethical approval from the NHS National Research Ethics Service (16/NW/0274). All participants gave informed consent for participation in UK Biobank. Permission to access and analyse UK Biobank data was approved under UK Biobank project 14151. SAIL analyses were approved by SAIL Information Governance Review Panel (Project 0830).” Page 16 lines 340-343

Results:
Please provide a table showing the baseline characteristics of the study population (Table 1).

We have added Table 1 to the manuscript giving descriptive baseline characteristics for each dataset.

Please note that figures and tables in the Supplementary Information cannot be grouped together in one document (<https://journals.plos.org/plosmedicine/s/supporting-information>). Please refer to a specific figure or table e.g. Figure S1, Table S1, etc. throughout the main text.

Thank you, we have uploaded these as requested.

Please report percentages to at least one decimal place.

These are now reported to one decimal place.

Please present numerators and denominators for percentages, at least in the Tables [not necessarily each time they're mentioned].

We now report denominators for all percentages.

Line 224 - please clarify what is represented by the numbers in brackets

These refer to 95% confidence intervals. We have added this to the text.

Where adjusted analyses are presented, please also provide the unadjusted analyses (this may be in  a Table) and please indicate which factors are adjusted for.

Thank you. For the adjusted hazard ratios/incidence rate ratios in figures 3b, 4b, 5b and 6 (now numbered 4, 6, 8 and 9) we have added supplementary tables with the unadjusted estimates. We have added to the figure legends what variables were adjusted for.

Where possible, please provide the actual numbers of events for the outcomes (this may be in  a Table), not just HRs.

We have added this information to the figures as requested.

Discussion:
Please present and organize the Discussion as follows: a short, clear summary of the article's findings; what the study adds to existing research and where and why the results may differ from previous research; strengths and limitations of the study; implications and next steps for research, clinical practice, and/or public policy; one-paragraph conclusion.
Please remove all subheadings within your Discussion e.g. Strengths and Limitations, Conclusion

Thank you. We have removed the subheadings as requested and ensured that the discussion is structured as requested.

Figures:
Please provide titles and legends for all figures (including those in Supporting Information files).
Please define all abbreviations used in the respective figure legend.

Figure legends are now detailed in the main text, and the supplementary figures at the end of the document.

Fig 3a, 4a - please indicate what is represented by the coloured areas and central lines in your graphs; 95% CI?

The central line shows the point estimate and the shaded area the 95% confidence interval. These are now specified in the figure legends.

Fig 3b - please clarify whether adjusted HRs are shown. Please indicate in the figure caption the meaning of the boxes and whiskers shown for UKBB ; why are these not provided for SAIL data?

These are adjusted hazard ratios. We now specify this in the figure legend. The box displays the point estimate and the whiskers the 95% confidence interval (now in the legend also). For SAIL, this was also the case but as the 95% confidence intervals were narrow, these were obscured by the box. We have adjusted the box-size to avoid obscuring the confidence intervals.

References:
Please ensure that journal name abbreviations match those found in the National Center for Biotechnology Information (NCBI) databases, and are appropriately formatted and capitalised.
Please also see <https://journals.plos.org/plosmedicine/s/submission-guidelines#loc-references> for further details on reference formatting.

References formatted accordingly.

Supplementary files:
Please provide titles and legends for each individual table and figure in the Supporting Information.
Please note that figures and tables in the Supporting Information cannot be grouped together in one document. Please see <https://journals.plos.org/plosmedicine/s/supporting-information> for our supporting information guidelines.

Thank you. We have formatted the supplementary figures as requested.

Comments from the reviewers:

Reviewer #1: "Associations between multimorbidity and adverse health outcomes: comparison of cohorts from UK Biobank and a representative community sample" compares two datasets: a UK Biobank cohort (n=211,597), and a Wales-based SAIL databank (n=852,055) covering some 70% of the Welsh population, for multimorbidity on n=40 long-term conditions (LTC). Various adjusted associations were examined with both unweighted and weighted (Cambridge) scores, and the datasets were found to be similar for associations between multimorbidity and various outcomes when LTC<=3, i.e. UK Biobank appears broadly representative for such cases. However, for LTC>3, UK Biobank estimates were found to likely be conservative. Some plausible explanations for the divergence in observed associations for LTC>3 were discussed.

The analysis appears generally well-executed, and should help to justify the validity of UK Biobank cohort data for research involving populations with relatively low multimorbidities. A number of comments follow:

1. It might be briefly commented as to whether the LTC definitions, and LTC classification by medical personnel, is likely to be applied very similarly for both UK Biobank and SAIL, i.e. are there any significant variances in medical practice between the component countries of the U.K.?

We used consistent definitions, using the same coding system, for both datasets. This was based on the Read coding system which is used in primary care throughout the UK and is consistent across the different nations of the UK as well as between primary care IT systems. We have added the following text to the methods to clarify this:

“Read codes are used in primary care to record diagnoses as well as coding events such as prescriptions. The coding system is common to Wales and the rest of the UK. LTCs were identified from Read codes occurring prior to the baseline assessment, using the criteria described by Barnett et al. For most conditions any diagnostic Read code occurring prior to baseline assessment was taken to indicate the presence of the LTC (as per the previous study on which the definitions were based).[25] For some pre-specified conditions the presence a recent diagnostic code (e.g. depression, anxiety) or of specific prescriptions dispensed in the year prior to baseline (e.g. asthma, constipation, pain, dyspepsia, migraine, epilepsy) were used to confirm if the condition were current (also according to definitions described by Barnett et al).[25] A list of all Read codes used for diagnoses and prescriptions is included in the supplementary material (S1 File and S2 File).

At the time of baseline assessment, many long-term conditions in the UK were managed and coded according to the Quality Outcomes Framework. These standards were applied in Wales similarly to the rest of the UK. Median time from practice registration to baseline assessment (from which diagnostic Read codes were assessed) was 14.6 years (interquartile range 7.4 to 23.3) in UK Biobank and 14.8 years (interquartile range 10.2 to 18.8) in SAIL.”

Pages 10-11, lines 186-201

2. It might also be commented as to whether there exists possible overlaps between UK Biobank and SAIL data, for Welsh participants, i.e. might some Welsh participants be considered as part of both datasets?

The reviewer is correct to point out that it is likely that some people in SAIL databank (given the criteria were inclusion was registration with a participating practice) were also UK Biobank participants. We have added the following to the limitations section:

“There may also be some overlap between UK Biobank participants in Wales and people included in SAIL databank, however we were unable to identify UK Biobank participants in SAIL.” Page 26, lines 589-591

3. Related to the above, it might be interesting to consider country-level sensitivity analyses if convenient/possible for UK Biobank - in particular UK Biobank (Wales) - vs. SAIL.

Thank you for this suggestion. We have added a sensitivity analysis comparing SAIL databank to UK Biobank participants from the Welsh assessment centres. This shows similar results to the analysis of the UK-wide analysis. We have added the following text and supplementary figures:

Methods:

“In sensitivity analyses, we repeated analyses of the effect of LTC count on mortality, hospitalisations, and MACE restricting the UK Biobank cohort to those participants who attended one of two assessment centres based in Wales. This was to assess if differences between UK Biobank and the community cohort were driven by differences between Wales and the rest of the UK. Separately, we also repeated the SAIL analyses restricting SAIL to participants registered in Cardiff and Swansea (the locations of the UK Biobank assessment centres in Wales). These analyses were not prespecified, being conducted following initial peer-review.” Page 16, lines 332-338

Results:

“In a sensitivity analysis of the relationship between LTC count and each outcome (all-cause mortality, hospitalisations, and MACE) we restricted UK Biobank participants to those attending assessment centres in Wales. The adjusted event rates were slightly lower in the Welsh UK Biobank subset than in the whole UK Biobank cohort (S35-37 Fig). Separately, we restricted the SAIL analyses to Cardiff and Swansea (the location of UK Biobank assessment centres), finding similar rates to the main analysis. This suggests that differences between SAIL and UK Biobank are unlikely to be driven by differences between Wales and the rest of the UK.” Page 21, lines 463-469

4. In Line 147, it is stated that "Age was taken as age at recruitment for UK Biobank, and calculated for 1st January 2011 for SAIL participants". It might be clarified as to whether the data involved must correspond to age at recruitment (for UK Biobank participants) or to 2011 (for SAIL participants); in particular, is it possible that relatively out-of-date data is involved in the analysis (e.g. SAIL participant had last check-up in say 2008, but those results are reported with his age in 2011)?

For UK Biobank, assessment of age, sex, socioeconomic status was based on the time of baseline recruitment, assessment of multimorbidity was based on Read codes occurring prior to that date, and all outcome assessment was based on events after the date of baseline assessment.

For SAIL, we mirrored this structure as closely as possible, taking 1^st^ January 2011 as the common ‘start’ date for all SAIL participants. Age calculated based on that date, multimorbidity assessed from prior diagnostic codes, and outcomes assessed prospectively.

The reviewer is correct that there may be a lag between the most recent primary care assessment and ‘baseline’, however the method of assessment is applied consistently to both datasets. In other words, multimorbidity assessment in UK Biobank for this study was based only on primary care codes and not on any assessment centre data. Therefore, the extent to which this data is ‘out of date’ is likely to be similar between both datasets. Importantly, we ensured that SAIL participants were known to be alive at the baseline date.

We have altered the following text to clarify this:

“Age was taken as age at baseline for both datasets (this corresponded to date of recruitment for UK Biobank, and 1^st^ January 2011 for SAIL participants).” Page 12, lines 225-226

5. In Line 172, it is stated that "We used fractional polynomials to model non-linear relationships between age and Townsend score and LTC counts". The specific methodology might be described in supplementary material and/or cited as appropriate.

Thank you. We have added the following text as well as a reference for the use of fractional polynomials.

“We used up to two fractional polynomial terms (from the set {−2, −1, −0.5, 0, 0.5, 1, 2, 3} recommended by Royston 1999) to model non-linear relationships between age and Townsend score and LTC counts. The best fitting fractional polynomials were identified using backward selection in the *mfp* package in R.” Page 13, lines 260-263

(reference added: Royston P, Ambler G, Sauerbrei W. The use of fractional polynomials to model continuous risk variables in epidemiology. International journal of epidemiology. 1999 Oct 1;28(5):964-74.)

6. In Line 184, it is stated that "Weibull models including fractional polynomials for age and Townsend scores, as well as interaction terms between LTC count and age, and LTC count and sex, were found to fit the data best". It might be clarified as to what this "best" is compared against.

We compared models using the Akaike information criterion (AIC) as well as comparing the likelihood ratio for nested models. We have expanded this section of the methods:

“Best fitting models were selected by comparing AIC and likelihood ratios.” Page 15 lines 297-298

7. In general, for the Statistical Analysis section, it might be considered to state the (Supplementary) Figure/Table corresponding to each analysis, and label the Supplementary Figures/Tables accordingly.

Thank you for this suggestion. We have added subheadings to the statistical analysis section, as well as similar subheadings to the results section (so that it is clear which analysis corresponds to which section of the results). We have not specifically indicated figures/tables in the methods section (as it is not standard to refer to results figures within the methods section), however if the editors wish us to add these to the methods we would be happy to do so.

8. For Figure 2, it might be illustrative to also display raw unadjusted counts for SAIL, if possible.

Thank you. We have added supplementary tables giving the unadjusted counts, as well as the expected counts based on standardisation.

Reviewer #2:
The study covers an important topic in multimorbidity research, with most likely implications for other cohort studies (also beyond the UK). The methods and results are well presented with systematic comparison with various methods and outcomes. I enjoyed reading the paper.

Major comments:
1) how did the authors handle differences in follow-up time, or completeness of follow-up across datasets? I can image that differences in attrition or (predicted) time-horizons may contribute to differences in absolute risks of adverse health outcomes over time.

Thank you for raising this point. We have changed our analysis to ensure similar follow-up times for both datasets. Follow-up data were available in SAIL from 1^st^ January 2011 to 1^st^ July 2018. UK Biobank follow-up was longer (and varied depending on the data source). In response to reviewer comments we have now censored UK Biobank follow-up at 7.5 years, death, or de-registration, whichever happened first.

We have updated all analyses, tables and figures with this new follow-up time in UK Biobank (equal to that in SAIL). The results are similar, and the interpretation unchanged. We have also added the following text to the methods:

“In SAIL, linked outcome data were available for 7.5 years follow-up (censored at 1^st^ July 2018). Participants were censored at date of death, re-registration from a participating practice, or 7.5 years follow-up, whichever occurred first. For UK Biobank, to ensure comparable follow-up periods for outcome assessment, we censored participants similarly at death, de-registration, or 7.5 years.” Page 12, lines 241-245

2) The authors restricted their analyses to a sample of 40 to 70 years old adults. Why was this done? (40-70 was baseline recruiting age for UK Biobank, but I suppose accrued follow-up now also allows for broader analyses on an age-scale)

The primary motivation for this work was to assess the utility and reliability of UK Biobank for multimorbidity research. Most UK Biobank studies use data from the baseline assessment centre (age range of eligibility 40-70) to assess exposures of interest and then follow participants up over time. We designed our analyses in a similar way (based on baseline age) to be similar in design to most UK Biobank analyses.

Our identification of participants in SAIL was based on a similar process, applying age-based eligibility criteria (albeit taking a single time-point for baseline).

We then applied our definition of exposure (multimorbidity) consistently to both datasets.

Our adjustment for baseline age is consistent with most published UK Biobank analyses.

Furthermore, the parametric survival models fitted to allow export from the SAIL secure platform and subsequent prediction of risk at varying covariate levels do not support ‘START STOP’ format survival data which would be required to model age as the time-scale.

3) I commend the authors with their great description of their sophisticated statistical analyses, that is provided in great detail.

Thank you

4) It may perhaps be useful to readers to know why a cut-off of 3 for LTCs was chosen to compare findings across studies?. Especially since absolute risks for mortality and hospitalization between the two datasets are systematically higher in SAIL compared to UK biobank, regardless of multimorbidity count. Addition of an analysis plan that describes these analyses would benefit the paper, as well as adhering to STROBE guidelines (including for example the checklist)

We did not set out to compare people with 0-3 LTCs with those with 4 or more. Rather, when assessing our results across the range of LTC counts observed in the data, the clearest divergence of relative risk of outcomes between SAIL and UK Biobank occurred at counts greater than three (hence the discussion of four of more).

The reviewer is correct, however, that this is a somewhat blurred distinction as absolute rates differ across all levels of multimorbidity and there are some differences in the pattern between outcomes. We have re-phrased the sections where this is discussed to make this clearer (quoted text below). We have also added our pre-specified analysis plan as a supplementary file.

“These findings suggest that, given its large sample size and extensive baseline assessment, UK Biobank is a valuable resource for exploring risks associated with chronic illness or multimorbidity with lower LTC counts. However, UK Biobank is likely to underestimate the impact of multimorbidity in people with greater numbers of LTCs or with specific LTCs such as mental health conditions. This difference was most obvious among participants with LTC counts or 4 or more.” Page 22, lines 481-486

“However, its lack of representativeness limits inferences about the minority of people with higher levels of multimorbidity, most noticeably those with LTC counts of 4 or more” Page 27, lines 601-602

5) what were reasons to predict the risk of MACE instead of the actual observation risk? Using the cumulative incidence function, it is perhaps more useful to simply show the observed (absolute) rates. Introducing the prediction term, introduces potential confounding of model misspecification or lack of generalizaiblity of the underlying predictive model.

Thank you for this comment. The motivation for modelling the risk of MACE rather than using observed rates was to maintain privacy. Analyses were required to be performed within a secure safe haven (the SAIL platform) and only non-disclosive results could be exported. This can be challenging for certain combinations of characteristics (eg those individuals with high numbers of comorbidities for a given age). It becomes a particular problem where cut-points need to be changed (eg different age-bands) as comparisons between different tables can be disclosive. Generally regression based outputs are less likely to result in disclosure than other data, and allows flexibility in terms of subsequent summaries (https://ukdataservice.ac.uk//app/uploads/thf_datareport_aw_web.pdf ). However, we fully agree that reviewers should be in a position to judge the appropriateness of our modelling choices. We have therefore exported coarsely-aggregated absolute event rates from the SAIL platform and have added these to the supplementary appendix (S3 Table). We have also incorporated these into the plots of the predicted rates to demonstrate the model fit (Supplementary figures S2 and S3).

6) in the methods section, SAIL is introduced as a representative sample of Wales. 70% of the population is covered. Can the authors provide some information on the missing 30% of people? Are they by any chance expected to be any different from the ones that are included?

The 70% included in SAIL are similar to the overall Welsh population in terms of age, sex and socioeconomic status. We have added a reference to our previous work demonstrating this, as well as adding the following text:

“Participating practices cover approximately 70% of the population of Wales and are representative of the wider population in terms of age, sex and socioeconomic deprivation.^22,23^ Comparison between the SAIL population and the overall population of Wales are reported elsewhere.^23^” Page 10 lines 176-179

The relevant data can be found here (from the reference given above).

https://static-content.springer.com/esm/art%3A10.1186%2Fs12916-019-1427-1/MediaObjects/12916_2019_1427_MOESM2_ESM.pdf

1) When presenting the prevalence of individual LTCs in UK Biobank and SAIL, the authors note that most LTCs were more prevalent in SAIL, but some LTCs (eg. Parkinsons' disease) were more common in UK Biobank. I'd be helpful to discuss possible explanations for this.

Thank you. We have added the text below to offer an explanation:

“It is also possible that, for some people, living with a condition may be a motivation for participation in UK Biobank. Furthermore, some LTCs have been noted to be more common in more affluent populations.[29] We speculate that this may be one possible explanation for the higher prevalence of some conditions (e.g. Parkinson’s disease) in UK Biobank compared to SAIL.” Page 24, lines 524-528

7) The authors hypothesise that LTCs in UK Biobank may be less severe or advanced which would explain the difference in risks of mortality / hospitalisation, but did not offer any explanation as to why LTCs in UK Biobank might be less severe. Since UK Biobank offers information about participants beyond the routine data, it might be possible to explore this further and try to support this hypothesis by assessing the severity of LTCs using those extra information.

We have added the following hypothesis to the discussion section to justify our speculation that UK Biobank participants may have less severe disease.

“This could occur if severe disease resulted in increased functional limitation which in turn reduced the likelihood they would volunteer to attend for UK Biobank assessment.” Page 23, lines 497-499

The use of UK Biobank variables to assess the severity of these long-term conditions is limited by the lack of similar measures in the community comparison (e.g. SAIL or other similar datasets). A detailed comparison of disease severity between UK Biobank and the general population is beyond the scope of this manuscript. Furthermore, for most conditions such comparisons cannot be made with routine data.

The following sentence in the discussion highlights this limitation:

“Unfortunately, it is usually not possible to estimate severity of LTCs using routine data alone.” Page 23, lines 502-503

8) In the supplementary results, the authors elegantly stratified for depreviation scores. It however seems that regardless of these scores, risks for adverse health outcomes are systematically higher for SAIL compared to UK Biobank participants. Could the authors speculate what drove these higher risks, apparently something beyond social-economic status, age or sex.

This is likely to reflect residual confounding. This may be due to differences in disease severity, to limitations in area-based measures of socioeconomic status, or other unmeasured factors. We have added the following text to highlight the severity issue:

“This could occur if severe disease resulted in increased functional limitation which in turn reduced the likelihood they would volunteer to attend for UK Biobank assessment.” Page 23, lines 497-499

In our opinion, the difference in absolute rates of adverse outcomes within strata of age, sex and socioeconomic status may also reflect a limitation of the area-level measures of socioeconomic status. Townsend scores, as used to quantify socioeconomic status in both datasets, are based on participant postcode. Within a given area (postcode) there is likely to be heterogeneity in individual markers of deprivation, health and wellbeing. Many of these individual factors (e.g. education, income, access to transportation, disability, social support) may influence the probability of participation in UK Biobank, but will not be captured by an area-specific measure of socioeconomic status. We have added the following text to the discussion to highlight this:

“Within a given area (within which individuals will be assigned the same value for socioeconomic status) there is likely to be variation in factors such as income, education, disability, and behaviours such as smoking and alcohol consumption. These factors may influence the likelihood of volunteering for studies such as UK Biobank, but may not be adequately reflected in an area-based measure of socioeconomic status.” Page 23, lines 506-510


Minor comments:
1) In the introduction section, the authors introduce the term collider bias. Not all readers may be familiar with this term, could the authors briefly explain this in the introduction section.

We have added the following text:

“In particular, it is argued that notwithstanding the large sample size and presence of participants with multiple LTCs, selection pressures within the sample may lead to collider bias, leading to biased associations between risk factors and outcomes. A collider is a variable which is causally influenced by two other variables.[18] For example, if people with multiple LTCs and people who are more likely to be hospitalised or die are less likely to be recruited into UK Biobank (i.e., a ‘healthy cohort’ effect), this could bias the association between LTCs and those outcomes observed in UK Biobank.” Page 7, lines 122-128

2) Figure 1 depicts the distribution of LTCs. The authors subsequently report that the mean is higher in SAIL than in UK-biobank. However, this distribution is highly skewed, and I would either suggest to transform it to a normal distribution or report median or range instead of mean.

Thank you. We now report the median instead.

3) Figure 2. The triangles and circles are relatively small compared to the bars, perhaps enlarge to increase readability.

We have edited this figure to display lines with error-bars. We also have added an additional file with the observed and expected values with confidence intervals.

4) can the authors clarify to readers what this score exactly means? "Cambridge mortality score of 12 [mean value in UK Biobank], predicted 5-year mortality was 2.2% (2.1 to 2.3) in UK Biobank and 4.0% (4.0-4.1) in SAIL, see supplementary appendix)." For example, what kind of combinations does one then have?

We have added some illustrative examples to the text in question:

“When using the mortality weightings from the Cambridge multimorbidity score, and thus considering the type as well as number of LTCs, the difference in absolute mortality risk between SAIL and UK Biobank persisted (e.g. for a 60 year old man with a Cambridge mortality score of 12 [mean value in UK Biobank, approximately equivalent to a diagnosis of alcohol problems: score 12.72, or the combination of diabetes, coronary heart disease and hypertension: combined score 12.36], predicted 5-year mortality was 2.2% (2.1 to 2.3) in UK Biobank and 4.0% (4.0-4.1) in SAIL, see supplementary appendix).” Page 18-19, lines 383-389

5) It would be very helpful to discuss the generalizability of the findings to other cohort studies with risk of selection bias, and its implications for multimorbidity studies in general.

Thank you for this suggestion. We have expanded the following section of the discussion to address this point.

“Potential for selection bias is a problem for cohort studies in general, not just UK Biobank. This study emphasises the importance of assessing population demographics compared to the general population and being mindful of the potential for estimates to be biased by a lack of representativeness. Our findings suggest that UK Biobank may be biased (and likely conservative) in making inferences for the population of people with multimorbidity involving high LTC counts. However, the impact of selection bias is unpredictable, and this pattern may not necessarily be the same in other cohorts susceptible to selection bias. In the absence of a direct comparison with a representative sample, investigators should be cautious in their interpretation of findings derived from highly selected cohorts. This suggests a need for future research cohorts to intensify efforts to target recruitment to ensure people with multimorbidity or living in areas of high socioeconomic deprivation are included.” Pages 25-26, lines 567-577

We have also added the following point to the ‘author summary’ which has been added to the beginning of the manuscript:

- “Ideally, future LTC and multimorbidity research should combine insights from both representative routine data and information rich research cohorts such as UK Biobank.”

6) The authors might consider adding confidence intervals to the modelled counts in Figure 2.

Thank you for this suggestion. We have now added these. The confidence intervals around these estimates are very narrow and do not visualise particularly well on the scale of the plot itself. We have added a table to the supplementary material with observed and expected counts from each model, along with 95% confidence intervals. This is now indicated in the figure legend.

7) the results section contains very valuable information, but also covers a lot of different aspects of the paper. Perhaps subheadings may help guide the reader through these different parts.
Thank you for this suggestion. We have added subheadings to the results section.

Thank you, Silvan Licher

Reviewer #3: Review
This is an interesting paper, but I feel the analysis could do with expanding (in terms of description, at least). I am also concerned by the fact that UKBB includes people from England, Wales and Scotland (but only those near the assessment centres), whereas SAIL includes participants from across Wales.

Main comments
1.      The comparison is between UK Biobank and a Welsh representative sample - so it is possible that differences between the rest of the UK and Wales could account for some/all of the differences observed here.

Thank you for this suggestion. We have added a sensitivity analysis which compared UK Biobank participants from Wales with the SAIL population. The following text has been added to the methods:

“In sensitivity analyses, we repeated analyses of the effect of LTC count on mortality, hospitalisations, and MACE restricting the UK Biobank cohort to those participants who attended one of two assessment centres based in Wales. This was to assess if differences between UK Biobank and the community cohort were driven by differences between Wales and the rest of the UK. This analysis was not prespecified, being conducted following initial peer-review.” Page 16, lines 332-338

This analysis showed that the rates of mortality, hospitalisation, and MACE, conditional on age, sex, socioeconomic status and number of LTCs, were marginally lower in the Welsh UK Biobank population than in the cohort as a whole. There was therefore a slightly greater difference between SAIL and UK Biobank (Wales) than between SAIL and UK Biobank as a whole. It is therefore unlikely that the findings we report are due to differences between Wales and the rest of the UK. We have added the following text to the results:

### “Sensitivity analyses

In a sensitivity analysis of the relationship between LTC count and each outcome (all-cause mortality, hospitalisations, and MACE) we restricted UK Biobank participants to those attending assessment centres in Wales. The adjusted event rates were slightly lower in the Welsh UK Biobank subset than in the whole UK Biobank cohort (S35-57 Fig). Separately, we restricted the SAIL analyses to Cardiff and Swansea (the location of UK Biobank assessment centres), finding similar rates to the main analysis. This suggests that differences between SAIL and UK Biobank are unlikely to be driven by differences between Wales and the rest of the UK.” Page 21, lines 463-469

2.      There were only two UKBB assessment centres in Wales, in Swansea and Cardiff. It would be useful to consider restricting the SAIL analysis to participants registered in Swansea or Cardiff, to ensure comparability.

As described in the response above, we have now included a sensitivity analysis restricting SAIL to people living in Cardiff or Swansea.

3.      "LTCs were identified from Read codes occurring prior to the baseline assessment, using the criteria described by Barnett et al." It would be useful to have more detail here, e.g. how long before the baseline assessment could the Read code occur? (there is presumably a cut-off?)? Was the % of participants with GP records going back 1,2, 5 however-many years the same across UKBB and SAIL? Any differences in READ recording between UK and UKBB? Could the list of READ codes be provided in supplementary material?

“We quantified multimorbidity using a count of 40 LTCs originally developed for a large epidemiological study in Scotland.[25] Read codes are used in primary care to record diagnoses as well as coding events such as prescriptions. The coding system is common to Wales and the rest of the UK. LTCs were identified from Read codes occurring prior to the baseline assessment, using the criteria described by Barnett et al. For most conditions any diagnostic Read code occurring prior to baseline assessment was taken to indicate the presence of the LTC (as per the previous study on which the definitions were based).[25] For some pre-specified conditions the presence a recent diagnostic code (e.g. depression, anxiety) or of specific prescriptions dispensed in the year prior to baseline (e.g. asthma, constipation, pain, dyspepsia, migraine, epilepsy) were used to confirm if the condition were current (also according to definitions described by Barnett et al).[25] A list of all Read codes used for diagnoses and prescriptions is included in the supplementary material (S1 File and S2 File).

At the time of baseline assessment, many long-term conditions in the UK were managed and coded according to the Quality Outcomes Framework. These standards were applied in Wales similarly to the rest of the UK. Median time from practice registration to baseline assessment (from which diagnostic Read codes were assessed) was 14.6 years (interquartile range 7.4 to 23.3) in UK Biobank and 14.8 years (interquartile range 10.2 to 18.8) in SAIL.” Pages 10-11, lines 185-201

4.      Similarly, "Finally, we identified combinations of three LTCs from previous studies of multimorbidity clusters,7,12 and assessed all possible combinations of the conditions within each cluster." - more information both in main text and as supplementary would be useful - I could not understand from this description what the outcomes were.

Thank you. We have expanded on this description of the analysis of combinations of specific LTCs:

“Finally, we identified combinations of three LTCs from previous studies of common co-occurring LTCs (clusters).^7,12^ We assessed the relationship between all possible combinations of the conditions within each cluster and all-cause mortality, unscheduled hospitalisation, and MACE. For example, for the cluster of coronary heart disease, diabetes and stroke we assessed the risk of adverse outcomes in each condition individually, in all pairwise combinations, and in people with all three conditions. The clusters of conditions assessed were: coronary heart disease, diabetes, and stroke or transient ischaemic attack; hypertension, diabetes and pain; pain, deafness and irritable bowel syndrome; depression, pain and anxiety; asthma, pain and chronic obstructive pulmonary disease; alcohol problems, substance misuse and pain; coronary heart disease, pain and depression.” Pages 11-12, lines 214-223

5.      For outcomes, what was the cut-off date in each dataset (i.e. censoring date)? Is length of follow-up comparable?

For SAIL we had follow-up data up until 1^st^ July 2018 (7.5 years from the beginning of follow-up).

We have now updated our UK Biobank analyses to have the same length of follow-up (i.e. participants were censored 7.5 years after baseline assessment, at death, or de-registration, whichever happened first).

We have updated all analyses with this amended follow-up time. Findings are similar, and all figures and tables have been updated accordingly. We have also added the following to the methods:

“In SAIL, linked outcome data was available for 7.5 years follow-up (censored at 1^st^ July 2018). Participants were censored at date of death, re-registration from a participating practice, or 7.5 years follow-up, whichever occurred first. For UK Biobank, to ensure comparably follow-up periods for outcome assessment, we censored participants similarly at death, de-registration, or 7.5 years.” Page 12, lines 241-245

6.      I found the description of the standardisation a little confusing. I am used to standardisation being either direct or indirectly standardised rates, i.e. here would have direct standardisation within strata of age/sex (with age grouped) and then within strata of age/sex/SES, comparing the number of cases (multimorbidity rates) in UKBB with the number expected from SAIL rates.  I think something similar is done here, except that a parametric model is used rather than strata and direct comparison of numbers of rates. More detail on how this model was derived would be helpful - i.e. exactly how were fractional polynomials used, what degree did you go up to, how did you assess model fit, etc. Did the negative binomial models fit well? Were any other models considered?
The fit of the models in SAIL is crucial to the interpretation of their fit to the UKBB data.
How did you estimate the confidence intervals for the UKBB estimates?

The motivation for using regression models in to compare SAIL and UK Biobank LTC counts was to maintain privacy. Analyses were required to be performed within a secure safe haven (the SAIL platform) and only non-disclosive results could be exported. This can be challenging for certain combinations of characteristics (eg those individuals with high numbers of comorbidities for a given age). It becomes a particular problem where cut-points need to be changed (eg different age-bands) as comparisons between different tables can be disclosive. Generally regression based outputs are less likely to result in disclosure than other data, and allows flexibility in terms of subsequent summaries (https://ukdataservice.ac.uk//app/uploads/thf_datareport_aw_web.pdf ).

We have re-written the description of this process as below:

### “Comparison of multimorbidity distribution

We first plotted the distribution of LTC counts in each dataset and compared these graphically to assess the observed distribution of multimorbidity in each dataset.

We then compared the distribution of LTC count in UK Biobank with SAIL after standardizing SAIL to the age/sex distribution, and then the age/sex/socioeconomic status distribution, of UK Biobank. To do this, we first modelled the distribution of LTC count in SAIL on age and sex, and on age sex and Townsend score. We assessed Poisson and negative binomial models initially. We used up to two fractional polynomial terms to model non-linear relationships between age and Townsend score and LTC counts. The best fitting fractional polynomials were identified using backward selection in the *mfp* package in R. We also explored interaction terms between each of the explanatory variables. Candidate models were assessed by comparing the likelihood ratio and assessing the dispersion statistic for each model. We also compared the best-fitting models to zero-inflated models using Vuong tests. Finally, we plotted expected and observed LTC counts within strata of age, sex and Townend score. The negative binomial model was found to fit the data well. We exported the model coefficients, variance-covariance matrix, and dispersion parameter from the secure platform, along with diagnostic plots summarising model fit (S1 Fig). This allowed us to model the expected distribution of LTC counts in SAIL conditional on age/sex and on age/sex/Townsend scores.

Next, we calculated the expected LTC count in UK Biobank based on the models fitted in the SAIL data. To do this, we aggregated the UK Biobank dataset by age (1-year bands), sex, and Townsend score (0.1-point intervals). We summed the total number of participants, as well as the number of participants with each LTC count, in each stratum. We used the coefficients of the negative binomial models exported from SAIL to calculate the expected mean LTC count for each stratum. Next, we used the cumulative density function for the negative binomial distribution to calculate the expected proportion of participants with each LTC count within each stratum. Finally, each expected proportion was multiplied by the number of UK Biobank participants in each stratum to obtain the expected number of participants with each LTC count. We then summed these expected values across strata to calculate the expected number of UK Biobank participants with each LTC count.

To calculate 95% confidence intervals for these estimates we obtained 10 000 samples of the intercept, age, sex and Townsend score coefficients by sampling from a multivariate normal distribution where the parameters were the point estimates and variance–covariance matrix for the SAIL Poisson regression models. We then repeated the above process with each of the 10,000 samples to obtain 10,000 estimates of the expected count, which we then summarised by the mean, 2.5th and 97.5th centiles to obtain 95% confidence intervals.” Pages 13-14 lines 254-288

7.      There is a similar lack of detail for the survival models. Weibull were found to fit best, but what other options were considered? What criteria were used to arrive at the best-fitting model? This information is unclear: "censoring participants dying of non-cardiovascular causes and coding as event status '0'". Was there an overall censoring date, and did it differ between participants (and was length of follow-up similar for SAIL and UKBB)?

We fitted parametric survival models as the SAIL analyses were conducted within a secure platform which only permits export of summary data. By modelling outcomes in this way, and exporting the model parameters (coefficients and variance-covariance matrix) we were able to produce model outputs for any given covariate level outside of the secure environment (as in the plots included in the paper and supplementary files).

As described in the response to comment 6, we have altered the UK Biobank follow-up time so that this is equivalent to the follow-up available in SAIL. We have added the following detail:

“In SAIL, linked outcome data was available for 7.5 years follow-up (censored at 1^st^ July 2018). Participants were censored at date of death, re-registration from a participating practice, or 7.5 years follow-up, whichever occurred first. For UK Biobank, to ensure comparable follow-up periods for outcome assessment, we censored participants similarly at death, de-registration, or 7.5 years.” Page 12, lines 241-245

We have added the following text to the methods section to describe the process of model selection more fully:

### “Modelling all-cause mortality and MACE

For all-cause mortality and MACE, we used parametric survival models to assess the relationship between LTC counts and each outcome. This allowed us to export model summary statistics from the SAIL secure platform which could facilitate estimation of predicted event rates for any combination of covariate levels. Before fitting models, we plotted log(time) against log(-log(Kaplan-Meier estimates)) for strata of each covariate. We found that for age (5-year bands), sex, and Townsend scores (1-point intervals) lines were linear and parallel, suggesting that Weibull models were an adequate fit for the data. Up to two fractional polynomial terms (selected using similar methods to the previous section) and interaction terms were explored. Best fitting models were selected by comparing likelihood ratios. We finally compared Weibull models to more flexible Generalised Gamma models. Weibull models including fractional polynomials for age and Townsend scores, as well as interaction terms between LTC count and age, and LTC count and sex, were found to fit the data best. We summarised the overall fit by plotting predicted and observed 5-year event rates (S2-3 Fig).” Pages 14-15, lines 289-302

8.      Why was Poisson used for unscheduled hospitalisation? What (exactly) was the outcome? Why was time to first unscheduled hospitalisation not used? Were there many people with multiple unscheduled hospitalisations and how were they dealt with? Was the assumption made that 1 person with 5 unscheduled hospitalisations over 5 years contributes the same information as 5 people each with 1 unscheduled hospitalisation over 1 year? I would think it was possible that there might be over-dispersion, or that some people may be prone to several unscheduled hospitalisations?

Hospital admissions in the UK are coded as emergency, urgent, or elective (with some regions using just two codes: urgent or elective). Our outcome of unscheduled hospitalisations was a count of all inpatient hospital episodes coded as emergency or urgent (i.e. non-elective). We have clarified this in the methods section:

“Unscheduled hospital admission was defined as any inpatient hospital episode coded as “urgent” or “emergency” (excluding admissions coded as “elective”).” Page 12, lines 238-239

This allowed us to take account of repeated events within individuals, and to offset by log(observation time).

We assessed model fit for the count models using a similar strategy to that of modelling number of long-term conditions (assessing observed and expected counts for candidate models, assessing dispersion statistics, and assessing for zero-inflation using Vuong tests). Prompted by the reviewer comments, we reviewed these models again in SAIL, and found that a negative binomial model had a closer fit to the data. As such we have revised our analysis to use a negative binomial model rather than Poisson. Plots showing model fit are shown below and in the supplementary files. This has not changed the interpretation of our results.

“For unscheduled hospitalization, we fit count-based models using a similar strategy that that describes above for modelling number of LTCs. Briefly, we compared Poisson and Negative Binomial models (with and without fractional polynomials and interaction terms) using likelihood ratios and dispersion statistics. We also assessed for zero-inflation using Vuong tests. Overall fit of the final model was assessed by plotting observed and expected numbers of hospitalisations for each cohort as a whole, and within strata of age, sex and socioeconomic status. Negative binomial models were found to best fit the data, and included non-linear terms for LTC count and age, as well as interactions between age and sex; age and LTC count; and sex and LTC count. Models for UK Biobank and SAIL, respectively, were used to assess hospitalization rates per 1000 person years at given levels of LTC count (plus age, sex, and socioeconomic status). Model fit is summarised in supplementary figures S4-5” Pages 15-16, lines 312-324

9.      Results - figure 1 -I would actually like to see a table for both figure 1 and 2 with observed and expected numbers in it, rates and their CIs. It is hard to compare rates on the figure when the prevalences are quite low.

For figure 1 we have added the percentages as text above each of the bars.

For figure 2 we have added a supplementary table with the observed UK Biobank counts, the expected counts from each model, and 95% confidence intervals.

10.     Results - "After additionally standardizing by socioeconomic deprivation (red circles), however, the expected LTC counts in SAIL". Are these the expected LTC counts in SAIL, or the expected counts in UKBB given the rates in SAIL? I had assumed the latter, given the description of the methods. Thus on Figure 2, I would expect to see observed UKBB counts, and UKBB expected given SAIL age/sex model and UKBB age/sex distribution, and UKBB expected given SAIL age/sex/SES model and UKBB age/sex/SES distribution. To me "SAIL age/sex standardised to UKBB" implies predicted SAIL counts given UKBB age/sex model and SAIL age/sex distribution.

Thank you for this comment. These are expected LTC counts in UK Biobank given the observed LTC counts (conditional on age, sex and socioeconomic status) in SAIL. We have amended the labelling of figure 2 to make this clearer.

11.     I would like to see confidence intervals on all figures. Would be useful also to see obs/expected (as in the more usual standardisation), especially for the higher counts of multimorbidity as the prevalences are so low it is hard to compare them on the figure.

Confidence intervals are indicated by the shaded areas in all line plots and by the error-bars in all forest plots (as well as being reported in the figure table). On reviewing these, it is clear that for some estimates with small confidence intervals the size of the point in the plots has obscured the confidence intervals.

We have amended the labelling of all figures to make this clear, as well as the point-size and resolution of all figures to ensure that confidence intervals are easily visible as well as being reported in the figure table.

12.     What was the age/sex/SES distribution of UKBB and SAIL? I would expect to see this in a table in the main paper.

Thank you. We have added table 1 to the main paper which includes these details.

13.     In figure 3a, is the absolute mortality risk shown for a particular age/SES group, or averaged over the population as a whole, or something else? I can't relate the text about a specific age group to the figure?

Thank you for this comment. This was the modelled mortality risk at the mean age of the UK Biobank sample (56.5 years) and at the UK national median Townsend score. We have added this information to the legend of figure 3a (as well as to figures 4a and 5a, which were modelled using the same approach).

14.     For figure 3b, would be better to have points not blocks and then show the confidence intervals for both SAIL and UKBB. The same for figure 4b and 5b. And figure 6.

Thank you for this comment. We have amended this figure to make the confidence intervals more readily visible.

15.     This sentence "Importantly, the rank-order of LTCs in terms of their mortality risk was broadly similar between the two datasets." - what does this mean? How do you judge "broadly similar"? If this is important, I'd expect to see a table/figure showing the rank-order of LTCs in terms of mortality in the two datasets and some sort of comparison. If this is about the HRs shown in Figure 6, then having figure 6 ordered either by SAIL or UKBB HR would help (am not sure what it is ordered by, is roughly mortality risk but doesn't seem to be exactly HR in either dataset?).

Thank you for this comment. On reflection, we have removed this reference to the ‘rank-order’ as it is based, as the reviewer highlights, on a visual inspection of figure 6 rather than on a formal analysis of the order.

Figure 6 (renumbered as figure 9) is ordered by the mean of the hazard ratios from both datasets, for each index condition. We have added this to the figure legend.

16.     Discussion - "than in a similarly aged representative UK sample from SAIL databank," - I thought the SAIL sample was only Welsh?

We have re-phrased this to:

“than in a similarly aged representative sample from Wales (SAIL databank),” page 22, lines 472-473

17.     "Moreover, although generally less prevalent in UK Biobank, many LTCs had a similar relative risk of mortality between the two datasets.". This is a bit vague. How many? How do you judge "similar"?

We have changed the text to the following:

“although generally less prevalent in UK Biobank, many common LTCs such as hypertension, chronic kidney disease, chronic obstructive pulmonary disease and coronary heart disease had a similar relative risk of mortality between the two datasets” Page 22, lines 479-481

For the conditions highlighted the point estimates are very close, and confidence intervals overlapping (e.g. hypertension 1.23 (1.21-1.26) in SAIL and 1.29 (1.23-1.36) in UK Biobank, chronic kidney disease 1.78 (1.72-1.84) in SAIL, 1.78 (1.54-2.05) in UK Biobank).

Later in the same paragraph we discuss that other conditions (e.g. mental health conditions) show wide differences in mortality risk between the two datasets.

18.     "However, UK Biobank is likely to underestimate the impact of multimorbidity in people with greater numbers of LTCs (e.g. 4 or more) ". I think care is needed here - in all cases the comparison is to having no LTCs - whereas the sentence implies that the comparison is limited to those having 4 or more LTCs. The issue is that the difference between predicted impact of LTCs in SAIl and UKBB increases as the number of LTCs increases.

Thank you. We agree with the reviewer. We have rephrased the following sections in light of these comments:

“These findings suggest that, given its large sample size and extensive baseline assessment, UK Biobank is a valuable resource for exploring risks associated with chronic illness or multimorbidity with lower LTC counts. However, UK Biobank is likely to underestimate the impact of multimorbidity in people with greater numbers of LTCs or with specific LTCs such as mental health conditions. This difference was most obvious among participants with LTC counts or 4 or more.” Page 22 lines 481-486

“However, its lack of representativeness limits inferences about the minority of people with higher levels of multimorbidity, most noticeably those with LTC counts of 4 or more” Page 27, lines 601-602

19.     Was ethnicity examined at all here? Was ethnicity similarly distributed across SAIL and UKBB?

Unfortunately we do not have data on ethnicity for SAIL databank. We have now made specific reference to this in the limitation section:

“As discussed above, we lacked data on the severity of the included LTCs (in both datasets), as well as data on ethnicity and broader lifestyle factors from SAIL.” Page 26, lines 591-592

20.     "SAIL includes data from Wales only, whereas UK Biobank comprises England, Scotland and Wales, although this is unlikely to have a substantial impact on our findings". This feels like quite a big claim to make with no evidence - you could compare the SAIL results to UKBB both only including people from Cardiff and Swansea - although numbers will be smaller, it would help to see how much the differences are due to Wales vs England vs Scotland?

We have added a sensitivity analysis comparing UK Biobank participants from Wales to the SAIL databank sample. The following text has been added to the results section:

### “Sensitivity analyses

In a sensitivity analysis of the relationship between LTC count and each outcome (all-cause mortality, hospitalisations, and MACE) we restricted UK Biobank participants to those attending assessment centres in Wales. The adjusted event rates were slightly lower in the Welsh UK Biobank subset than in the whole UK Biobank cohort (S35-37 Fig). Separately, we restricted the SAIL analyses to Cardiff and Swansea (the location of UK Biobank assessment centres), finding similar rates to the main analysis. This suggests that differences between SAIL and UK Biobank are unlikely to be driven by differences between Wales and the rest of the UK.” Page 21, lines 463-469
