# Supplementary material for: Associations between multimorbidity and adverse health outcomes in UK Biobank and the SAIL Databank: A comparison of longitudinal cohort studies
Source: PLoS Med. 2022 Mar 7;19(3):e1003931. doi: 10.1371/journal.pmed.1003931 (PMC8901063; doi:10.1371/journal.pmed.1003931)
Supplement: Attachment — Submitted filename: PLOS medicine reviewer response round 2.docx [file pmed.1003931.s048.docx]

**Response to reviewers’ comments**

Thank you for the opportunity to revise this manuscript. Responses to each of the reviewers’ comments, along with any changes to the text, are detailed below.

**Reviewer #1**:

We thank the authors for addressing our previous comments, and have no further major issues to report. It may however be considered to further account for the significant difference in prior socioeconomic status distribution between SAIL and UK Biobank from Table 1 (which moreover might be expected to be 20% for each quintile, in a truly representative sample), if possible.

***Author response***

Thank you for this comment. We have added the following text alongside table 1:

“UK Biobank had a greater proportion of female participants. UK Biobank participants also tended to be more affluent (42.0% in the most affluent quintile ok the UK as a whole) whereas SAIL participants were on average less affluent than the UK population as a whole (10.6% in the most affluent quintile).”

**Reviewer #3:**
**Comment** **1**.

I think the abstract should mention that UKBB is UK-wide (2 centres in Wales) and that the SAIL is Wales.

***Author response:***

Thank you.

We have added these details to the description of the datasets in the abstract (added text in blue):

“These are observational analyses of cohorts identified from linked routine healthcare data from UK Biobank participants (n=211,597 from England, Scotland and Wales with linked primary care data, age 40-70, mean age 56.5 years, 54.6% women, baseline assessment 2006-2010) and from the Secure Anonymised Information Linkage (SAIL) databank (n=852,055 from Wales, age 40-70, mean age 54.2, 50.0% women, baseline January 2011).”

**Comment 2**.

Also can you clarify in the abstract why UKBB has half the expected number of participants? (I think because of linkage, but it would be useful to have this info in the abstract).

***Author response***

We have added to the abstract that the UK Biobank sample was those with linked primary care data (changes to text quoted above).

**Comment 3.**    Abstract - I don't think UKBB accurately estimates risks (because you say absolute risks are underestimated), but relative risk/risk ratio/hazard ratio?

***Author response***

We have edited the following sentence:

“In this study, we observed that UK Biobank accurately estimates relative risk of mortality, unscheduled hospitalisation, and MACE associated with LTC counts ≤3.”

**Comment 4**

Figure 2 could have confidence intervals on the expected number? I would expect CIs to be in the vertical plane not the horizontal. Also it would be useful if the legend for "expected" indicated that this was expected given the model in SAIL.

***Author response:***

As the reviewer highlights in the comment below, the confidence intervals around the expected count are very narrow. This is because they are based on the very large numbers of observations in the SAIL sample. Such narrow confidence intervals have been challenging to visualise (these are displayed on the current figure but are so narrow that the width of the error bar is more prominent – hence the appearance of the horizontal plane). We have emphasised in the figure legend that the confidence intervals are displayed in table form in the supplementary material. We also highlight that the expected count was expected given the model in SAIL:

“The blue line shows the expected LTC counts in UK Biobank based on the age and sex adjusted regression models fitted in SAIL. The red line shows the expected LTC counts in UK Biobank based on age, sex and socioeconomic status adjusted regression models fitted in SAIL. Observed and expected counts, with 95% confidence intervals, are also shown in S2 Table.”

**Comment 5.**

Supplementary Table 2 indicates that once SES is taken into account, there seem to be more people in UKBB with the high number of morbidities, compared to what would be expected from SAIL. The confidence intervals also look very narrow - though I appreciate this could be due to rounding to the nearest whole number?

***Author response:***

Thank you for this comment. The confidence intervals are so narrow because of the very large numbers of observations in the SAIL sample. We also agree that, at high levels of multimorbidity, the observed number in UK Biobank is slightly higher than the expected count after adjusting for number of morbidities, although there are still lower than expected levels of multimorbidity at counts of 2-5 (where the vast majority of multimorbidity exists, in absolute terms).

**Comment 6.**

"We then compared the distribution of LTC count in UK Biobank with SAIL after standardizing SAIL to the age/sex distribution, and then the age/sex/socioeconomic status distribution, of UK Biobank.". I found this description a bit confusing. My reading of the rest of the methods is that you fitted a model in SAIL, then used it to predict outcomes in UK Biobank. I don't see how this means that SAIL was standardized to the age/sex/SES distribution of UK biobank? I would have described it as the expected number in each LTC category if the rates in SAIL applied in UKBB? But this may just be semantics and the description of the methods is clear.

***Author response:***

We chose the term “standardisation” because, like the “indirect standardisation” procedure used in descriptive epidemiology, rates from a standard population (in this case SAIL) are applied to the age-sex-SES distribution of the population of interest (in this case UKBB) to obtain some expected value which is then compared to the observed values.  We appreciate that it is more usual in standardisation to use stratum-specific rates than model-based rates which  (in order to avoid selecting cut-points which raises issues of disclosiveness and can reduce the flexibility of the findings) we used here. We also appreciate that it is more usual in indirect standardisation to produce a single standard count (eg a mean comorbidity count) rather than a distribution of counts (number of individuals with each comorbidity count). However, we were unable to think of a better term that could be used as short-hand to describe the method to a clinical/epidemiological audience. We therefore propose using the description “a form of standardisation analogous to indirect standardisation” rather than “standardisation” per se the first time we use this term in the manuscript.

We thank the reviewer for their comment that the description of the method was clear. We have edited the text that precedes it to the following:

“We then compared the distribution of LTC count in UK Biobank with SAIL after standardizing SAIL to the age/sex distribution, and then the age/sex/socioeconomic status distribution, of UK Biobank using a form of standardisation analogous to indirect standardisation. To do this, we first modelled the distribution of LTC count in SAIL…”

**Comment 7.**

Thanks for the sensitivity analyses restricting to the populations of Cardiff and Swansea. For mortality I agree that the main results are unaffected - but for unscheduled hospitalisations, the results appear much closer in the sensitivity analysis,

***Author response:***

Thank you for this comment. They are somewhat closer, although for most of the range of multimorbidity the UK Biobank (Wales) rate is lower than the overall UK Biobank rate, and the SAIL (Cardiff and Swansea) rate is lower than the overall SAIL rate, with a similar overall difference between the two. We have added the following text to highlight that the results of the sensitivity analysis are closer but that rates in SAIL remain higher than in UK Biobank.

“Separately, we restricted the SAIL analyses to Cardiff and Swansea (the location of UK Biobank assessment centres), finding similar rates to the main analysis for mortality and MACE. For hospitalisation, the difference between SAIL participants from Cardiff and Swansea and UK Biobank participants from Wales was less than the difference between each of the full cohorts (S36 Fig), although rates in SAIL remained higher than UK Biobank.”
